# Proteomics and Machine Learning–Based Approach to Decipher Subcellular Proteome of Mouse Heart

**DOI:** 10.1016/j.mcpro.2025.100952

**Published:** 2025-03-18

**Authors:** Haoyun Fang, Alin Rai, Seyed Sadegh Eslami, Kevin Huynh, Hsiao-Chi Liao, Agus Salim, David W. Greening

**Affiliations:** 1 Baker Heart and Diabetes Institute, Melbourne, Victoria, Australia; 2 Baker Department of Cardiometabolic Health, University of Melbourne, Parkville, Victoria, Australia; 3 Baker Department of Cardiovascular Research Translation and Implementation, La Trobe University, Bundoora, Victoria, Australia; 4 Faculty of Medicine, Nursing and Health Sciences, Monash University, Clayton, Australia; 5 School of Mathematics and Statistics, The University of Melbourne, Parkville, Victoria, Australia; 6 Melbourne School of Population and Global Health, University of Melbourne, Parkville, Victoria, Australia

**Keywords:** cellular organelles, heart, machine learning, proteomics, subcellular

## Abstract

Protein compartmentalization to distinctive subcellular niches is critical for cardiac function and homeostasis. Here, we employed a rapid and robust workflow based on differential centrifugal-based fractionation with mass spectrometry–based proteomics and bioinformatic analyses for systemic mapping of the subcellular proteome of mouse heart. Using supervised machine learning of 450 hallmark protein markers from 16 subcellular niches, we further refined the subcellular information of 2083 proteins with high confidence. Our data validation focused on specific subcellular niches such as mitochondria, cell surface, cardiac dyad, myofibril, and nuclear, unfolding dominant subcellular localization of proteins in their native environment of mouse heart. We further provide targeted nuclear enrichment and co-immunoprecipitation–based proteomic validation from the heart of nuclear-localizing protein networks. This study provides novel insights into the molecular landscape of different subcellular niches of the heart and serves as a draft map for heart subcellular proteome.

## Highlights

• Protein compartmentalization to subcellular niches is fundamental for cardiac function and homeostasis.
• Subcellular fractionation and MS-based proteomics provides comprehensive maps of spatial regulation of cardiac protein networks.
• Identify high confidence subcellular localization clusters for heart proteome.
• Validate subcellular proteome of distinct cardiac-specific subcellular niches.
• Draft map for mouse heart subcellular proteome.

## Graphical Abstract

**Figure:**
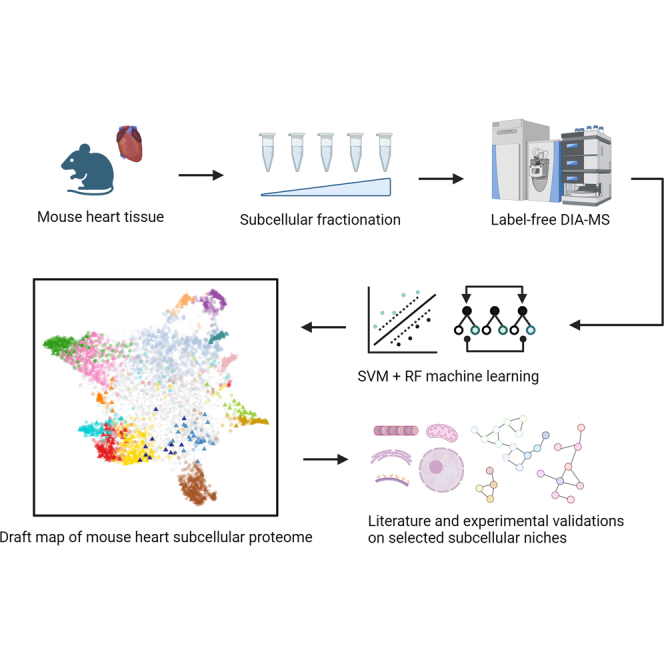

## In Brief

Subcellular localization of cardiac cell organelles is crucial for heart function. Mass spectrometry–based proteomics in combination with subcellular fractionation provides comprehensive maps of spatial regulation of protein networks and crucial insights in understanding cardiac physiopathology. Here, we provide a reproducible approach to fractionate, define, and validate the subcellular proteome of mouse heart in its native state. This study provides a resource of the proteome landscape of subcellular niches of the mouse heart and will serve as a draft map for heart subcellular proteome.

The heart is a highly structured and metabolic active organ that supports unidirectional blood flow via rhythmic contraction and dilation. Protein compartmentalization to different subcellular niches is critical for cardiac function and homeostasis (1, 2, 3). Intricate communication between subcellular organelles in the heart is essential for cardiac functions, such as calcium handling and cellular metabolism (4, 5, 6, 7). Structurally compromised and functionally defective cardiac organelles are associated with impaired cardiac performance and implicated in different cardiovascular diseases including congestive heart failure, ischemia cardiac injury, diabetic cardiomyopathy, and pathological cardiac hypertrophy (8, 9, 10, 11). For instance, perturbed cardiac mitochondria is linked to insufficient ATP production (12, 13), aberrant production of reactive oxidative species (14, 15), and impaired iron and calcium ion balance (16, 17). Endoplasmic reticulum (ER) or sarcoplasmic reticulum stress is associated with inflammation (18, 19, 20), lipotoxicity (21), and apoptosis (20) of cardiac cells. Therefore, understanding the molecular compositions of cardiac organelles is crucial for studying physiopathology of various cardiovascular diseases and identifying novel therapeutic targets (22). Biochemical-based organellar enrichment (23, 24) or proximity labeling–based tagging (25, 26) with mass spectrometry (MS)-based proteomics have been employed to identify specific subcellular organelles of interest. While these studies have provided valuable insights into specific organelle biology, the systemic construction of different subcellular niches occupying the cardiac landscape remains a challenge (27). Such a systems approach will provide a comprehensive overview of the spatial dynamics of cardiac proteins and their subcellular distribution in health and disease.

Over the last 2 decades, several proteomic pipelines have been established to ascertain subcellular distribution of different proteins in high-throughput, which include correlation profiling-based subcellular proteomics pipelines including protein correlation profiling (28), localization of organelle proteins by isotope tagging (29, 30), dynamic organellar maps (31, 32, 33), and SubCellBarCode (34). These strategies typically involve subcellular fractionation of cells or tissues using differential centrifugation or density gradient separation, followed by MS-based proteomics profiling. Based on quantitative proteomic data, machine learning (ML)-based approaches are applied to classify protein subcellular locations based on quantitative fractionation profiles of known organelle protein markers.

Here, we report a streamlined proteomics and bioinformatics pipeline for studying the subcellular protein composition of the mouse heart. Using low sample input (40 mg) of mouse heart tissue, we applied 10-step (11 fractions) differential centrifugation–based fractionation coupled with data-independent acquisition (DIA) MS for systemic mapping of the heart. This strategy revealed quantitative fractionation profile of 5134 protein groups. For subcellular protein assignment, we included 450 protein markers associated with 16 different subcellular niches. We then employed support vector machine (SVM), random forest (RF), and extreme gradient boosting (XGB) ML algorithms along with gene ontology (GO)-based and STRING database–based organellar specific inclusions to map the subcellular location of 2083 proteins with high confidence. Our study therefore provides a systems-level overview of subcellular proteome of the mouse heart with high resolution.

## Experimental Procedures

### Experimental Design and Statistical Rationale

In this study, we aimed to apply a reliable subcellular proteomics pipeline and construct a subcellular proteome map from heart. For generating subcellular proteomic data, we used frozen mouse heart tissue from six different mice (six biological replicates) and divided into two interday experiment sets of triplicates. For each replicate, 11 subcellular fractions (fraction) were prepared along with a whole heart homogenate (global) sample for single-shot DIA-MS (fraction: 66 MS files; global 6 MS files). For generating targeted nuclear-enriched proteomic data, we used frozen heart tissue from five mice (five biological replicates), further separated into two nuclear-enriched fractions (NUA, NUB) and one cytoplasmic fraction (CYT). For generating co-immunoprecipitation (Co-IP) MS data, we employed frozen heart tissue from three mice (biological replicates). We obtained the elute (EL) and unbound (UB) fractions of each target (antibody: Tnnt2, Csnk2a2, and IgG control) from Co-IP experiment for proteomic profiling (18 MS files).

For subcellular proteomic experiment data, we conducted coefficient of variation, Pearson correlation analysis, and nonlinear iterative partial least squares principal component analysis (PCA) to evaluate the variations between replicates, overall data quality, and the effect of missing value imputations. For targeted nuclear-enrichment proteomics and Co-IP MS experiments, we performed one-way ANOVA and student’s *t* test with Benjamini–Hochberg for multiple-testing correction. Adjusted *p*-value (Benjamini–Hochberg-corrected) < 0.05 and |log2 fold-change|>1 is deemed statistically significant.

### Heart Tissue Sourcing

Mice experiments and access to heart tissue carried out in this study were conducted in accordance with the guidelines and approval of the Alfred Research Alliance Animal Ethics Committee, Vic, Australia (ethics approval number, P2580). WT C57BL/6 mice were sourced from AMREP AS Pty Ltd, VIC. Hearts were isolated immediately following euthanasia and washed briefly in ice-cold PBS (manual perfusion) followed by snap-freezing in liquid nitrogen.

### Heart Subcellular Fractionation

Tissue homogenizations and subcellular fractionations were prepared as described with modifications (29). Intact frozen mouse hearts were individually ground as whole under liquid nitrogen using prechilled pestle and mortal, and sub-aliquots of ground tissue were transferred into 1.5 ml microtubes with prechilled 3.2 mm Stainless Steel Beads (Next Advance) with 500 μl of hypotonic lysis buffer (0.25 M sucrose, 10 mM Hepes pH 7.4, 2 mM EDTA, 2 mM magnesium acetate tetrahydrate) with HALT protease and phosphatase inhibitor (Thermo Fisher Scientific). Heart extracts were homogenized using prechilled Bullet Blender (Next Advance) at setting 10 for 15 s and setting 3 for 15 s and subsequently gently mixed in a rotary tube mixer for 10 min at 4 °C. Heart extracts were centrifuged at 200*g* for 5 min with three repetitions to remove tissue and cell debris. The supernatant was collected for the collection of whole heart homogenate (global) and the subsequent subcellular fractionations (fraction) using differential centrifugation. The subcellular fractionation protocol was performed using 40 mg of heart extract in a fixed-angle rotor centrifuge Eppendorf 5804R or Optima MAX-XP with TLA-55 rotor (Beckman Coulter), at 4 °C. Samples were initially centrifuged at 600*g* at 4 °C for 10 min before supernatant recovered and transferred to a clean tube; pellets following each centrifugation stage are labeled as fraction 1 (F01), sequentially to fraction 10. For each subcellular fraction, pellets were obtained following 1000*g* 10 min (F02), 3000*g* 10 min (F03), 5000*g* 10 min (F04), 9000*g* 15 min (F05), 12,000*g* 15 min (F06), 15,000*g* 15 min (F07), 30,000*g* 20 min (F08), 79,000*g* 43 min (F09), and 120,000*g* 45 min (F10). The final supernatant was collected and labeled as F11.

The enrichment of nucleus from frozen mouse heart was performed using NE-PER nuclear and cytoplasmic extraction reagents as instructed by the manufacturer (78833, Thermo Fisher Scientific). All fractions were stored at −80 °C before proteomics analysis. Protein quantitation was performed using microBCA assay (23235, Thermo Fisher Scientific).

### Western Blot Analysis

Western blotting was performed on samples (7.5 μg) as previously described (35). Rabbit antibodies raised against anti-RAB7 (Abcam, ab77993), anti-COX IV (3E11) (Cell Signalling Technology, 4850), anti-Calreticulin (Cell Signalling Technology, 12238), mouse anti-Cardiac Troponin T (BD Bioscience, 564766), and anti-GAPDH (D4C6R) (Cell Signalling Technology, 97166) were used. Secondary antibodies include IRDye 800 goat anti-mouse IgG (LI-COR Bioscience, 926-32210) or IRDye 680 goat anti-rabbit IgG (LI-COR Bioscience, 926-68071).

### Co-IP for Proteomics Analysis

For Co-IP experiment, anti-Troponin T-C antibody (CT3) (Santa Cruz Biotechnology, sc-20025) and anti-CSNK2A2 antibody (Abcam, ab10474) were used to identify binding partners of each target and anti-IgG antibody (Cell Signalling Technology, 2729) as negative control. Heart homogenate was prepared as described (above), with modification including preparation of the lysis buffer using 10 mM Hepes, 10 mM potassium chloride, 0.1% Triton X-100 with HALT protease and phosphatase inhibitor (Thermo Fisher Scientific), pH 7.4. Co-IP was conducted as described with modifications (36). We used 0.1% Triton X-100 instead of 0.05% NP-40 for preparing lysis buffer and 0.05% Triton X-100 (instead of 0.05% NP-40) for beads washing buffer and conducted antibody-antigen incubation for 18 h at 4 °C.

### Proteomics Sample Preparation

Subcellular fractions, whole heart homogenate, and Co-IP samples (3 μg protein) were denatured, reduced, and alkylated with 2% w/v SDS, 50 mM Hepes pH 8 (with HALT protease and phosphatase inhibitor), 10 mM DTT, and 20 mM IAA as described (37). Briefly, samples were prepared using SP3 protocol (38) and digested using Trypsin and Lys-C (1:50 and 1:100 enzyme-to-protein ratio, respectively) overnight at 37 °C. Samples were acidified after digestion to final concentration of 2% formic acid (FA) before vacuum lyophilization. Samples were reconstituted in 10 μl of 0.07% (v/v) TFA in LC-MS grade water; peptides quantified using fluorometric peptide assay (23290, Thermo Fisher Scientific).

### Liquid Chromatography and DIA MS

LC-MS data acquisition was performed on Q Exactive HF-X benchtop Orbitrap mass spectrometer coupled with UltiMate NCS-3500RS nano-HPLC and operated with Xcalibur software as previously described (37). Peptides (350 ng) were bound to a trapping column (Acclaim PepMap100 C18 3 μm beads with 100 Å pore-size, Thermo Fisher Scientific) in buffer A (100% LC-MS grade water, 0.1% FA) at 5 μl/min for 5 min in 55 °C and separated by analytical column (1.9-μm particle size C18, 0.075 × 250 mm, Nikkyo Technos Co. Ltd) with a scheduled gradient of 2 to 28% buffer B (100% acetonitrile, 0.1% FA) for 40 min, 28 to 80% for 2 min at 300 nl/min in 55 °C (butterfly portfolio heater, Phoenix S&T). MS1 full scan was set to 60,000 resolution, 3e6 AGC target, and maximum IT of 50 ms in 350 to 1100 m/z scan range. MS2 was set to 15,000 resolution, 1e6 AGC target, and 27 ms maximum IT. A total of 38 scan windows with staggered 20 m/z isolation window from 350 to 1100 m/z with 28% normalized collision energy. MS-based proteomics data is deposited to the ProteomeXchange Consortium via the MassIVE partner repository and available via MassIVE with identifier (MSV000096615).

### MS Data Processing and Analysis

DIA-MS spectra were processed using DIA-NN software (39) (v1.8) as previously reported from our lab (37, 40). In brief, DIA-MS spectra were searched against mouse proteome databases (UP000000589, #55,319, June 2022) retrieved from UniProt website (41). For library-free searches, “FASTA digest for library-free search/library generation” and “Deep learning-based spectra, RTs and IMs prediction” were selected, and matching between runs function was enabled. Trypsin/P was selected for enzymatic digestion with maximum 1 missed cleavage. The precursor change range was set to 1 to 4, and the m/z precursor range was set to 300 to 1800 for peptides consisting of 7 to 30 amino acids with N-term methionine excision and cysteine carbamidomethylation enabled as a fixed modification and variable (no variable modification) modifications were kept as default. Mass accuracies at MS1 and MS2 were automatically determined by DIA-NN via default setting (Mass accuracy: 0.0). Neural network classifier was set to single-pass mode. The mass spectra were analyzed using default settings with a false discovery rate (FDR) of 1% for precursor identifications.

To include proteins with high quantitative confidence, we include protein groups quantified in at least two out of three replicates from the same subcellular fraction from both experiment sets. For proteomic data normalization, we followed a similar data processing pipeline as described in Čuklina *et al.* (42). In brief, protein group label-free quantification intensities from both global and subcellular fraction proteomics datasets were normalized via variance stabilizing normalization (VSN) using normalizeVSN() function from limma package (43) in R. Batch adjustment of interexperimental proteomics data was completed using removebatcheffect() function from limma package. Missing values were imputed for each fraction using a modified two-step imputation approach (44); missing values at random imputed using neighbor-based K-nearest neighbors and missing values not at random imputed with left-censored approach (QRILC) using imputeLCMD package (https://CRAN.R-project.org/package=imputeLCMD). For downstream analyses, protein group intensities were normalized to sum intensity ratio (0–1) across 11 fractions of each experiment as described (31).

### ML-Based Classification of Subcellular Protein Localization

ML-based classification of subcellular protein localization was performed using R, applying pRoloc and pRolocextra packages (45, 46). For the training dataset, 450 manually curated protein markers were included to cover 16 subcellular categories with at least nine protein markers included per category (Supplemental Table S10). The protein markers were selected based on their subcellular annotation from multiple data sources including previous publications (29, 47, 48) and public databases (49, 50, 51, 52). For ML-based classification, we incorporated three independent algorithms, including SVM, RF, and XGB. To optimize different parameter combinations of each algorithm, we split marker protein sets to 0.8/0.2 training and validation set for each subcellular category. Parameter combination (cost and sigma for SVM, mtry for RF, max_depth and gamma for Xgboost) was optimized based on 5-fold cross validations on the training set (Supplemental Table S12), with the best combination selected by macro-F1 score. The optimized classifier was trained on the training set and evaluated on the validation set to yield a macro-F1 score, repeated 100 times to estimate generalization performance. We selected overall best performing parameters of each algorithm based on the best macro F1-score from different parameter combinations (Supplemental Table S13). The best parameters used were sigma at 0.01 and cost at 4 for SVM, mtry at 4 for RF, gamma at 3.2, and max depth at 6 for XGB (Supplemental Fig. S3, *B*–*D*, Supplemental Tables S12, S13). To obtain high confidence subcellular protein assignment, we applied a subcellular category-specific threshold as reported in Geladaki *et al*., (29) with minor modifications, to determine protein groups to be classified with high confidence. In detail, we prepared a binary column annotating 1) whether the predicted protein subcellular location can be found with matching GO annotation simplified in SubcellulaRVis (53) and 2) whether the predicted protein is a neighbor of another protein from the same subcellular category predicted from SVM, RF, and XGB using the information from STRING database (54) (Supplemental Table S16). From each model, proteins of each subcellular category were ranked in descending order based on their probability scores and a 5% FDR applied on the binary column to ensure a high confident subcellular protein prediction threshold.

### Bioinformatics Analysis

Proteome data analyses were processed using R (v4.2.3). Upset plot was generated via UpSet() function from ComplexHeatmap package (55), with waterfall plot, bar charts, profile plots, and boxplots generated using ggplot2 package (56). PCA was commenced using prcomp() function. Pearson correlation analysis of proteomes for each fraction was performed using the cor() function from stats package (https://www.R-project.org/). Hierarchical cluster analyses were performed using pheatmap() function from ComplexHeatmap package. For k-means clustering, we optimized the number of clusters using fviz_nbclust() function from factoextra package (https://CRAN.R-project.org/package=factoextra) and performed k-means clustering using kmeans() function from stats package. UMAP plot was generated using umap() function with n_neighbours = 50 and min_dist = 0.3 via umap package (https://CRAN.R-project.org/package=umap). F1 scores and quadratic losses were calculated as described (57, 58). GO-based functional enrichment analysis was performed using compareCluster() function from clusterProfiler package (59). Enrichment map was generated using emaplot() function from enrichplot package (https://doi.org/10.18129/B9.bioc.enrichplot). Selected protein network clusters visualized using Cytoscape software with STRING enrichment via StringApp (60).

## Result

### Subcellular Proteomics Pipeline for Frozen Heart Tissue

**Fig. 1:**
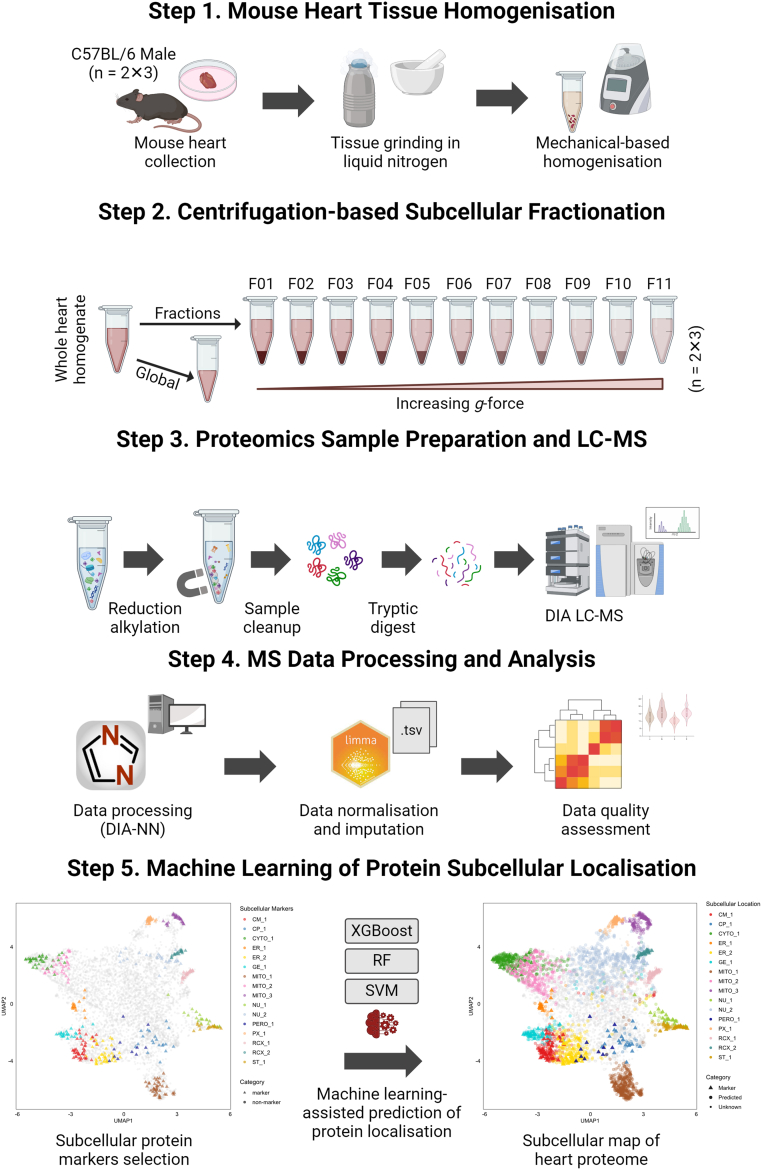
**Experimental workflow of subcellular proteomics of mouse heart.** Mouse hearts were isolated, washed in ice-cold PBS followed by snap-freezing in liquid nitrogen, and manual tissue disruption performed. Solubilized tissue homogenates were generated using hypotonic lysis and mechanical dissociation. Individual heart homogenates (total N = 6; two experimental sets of N = 3) were analyzed as whole heart homogenates (global) or subcellular fractionation workflow using sequential differential centrifugation (from 600*g* to 120,000*g*; F01-F11). Conventional SP3-based proteomic sample preparation, combined with DIA-MS were employed before data processing and informatics, including different machine learning–based algorithms for subcellular proteome annotation (RF, SVM, and XGB).

Here, we present a sequential fractionation and analytical pipeline for the comprehensive analysis of distinct subcellular compartments of mouse heart (Fig. 1). Briefly, frozen hearts (N = 6) were dissociated and subjected to homogenization (40 mg of heart tissue) (step i). Samples were subjected to differential centrifugation–based fractionation adapted from subcellular proteomics pipeline of cells (29, 31, 36) (step ii). Once subcellular fractions were collected, samples were lysed in SDS and reduced/alkylated before being subjected to protein hydrophobic and hydrophilic capture–based tryptic digestion (38) and LC-MS analysis. Proteins were subjected to direct DIA MS combined with label-free quantitation (31, 37) (step iii). Proteomic data was processed through DIA-NN and analyzed for reproducibility and quality assessment (step iv). Our workflow has further applied different ML approaches from pRoloc and pRolocExtra package suit (45, 61) to classify protein subcellular location (step v).

### Differential Centrifugation Enables In-Depth and Reproducible Subcellular Proteome Profiling of the Mouse Heart

**Fig. 2:**
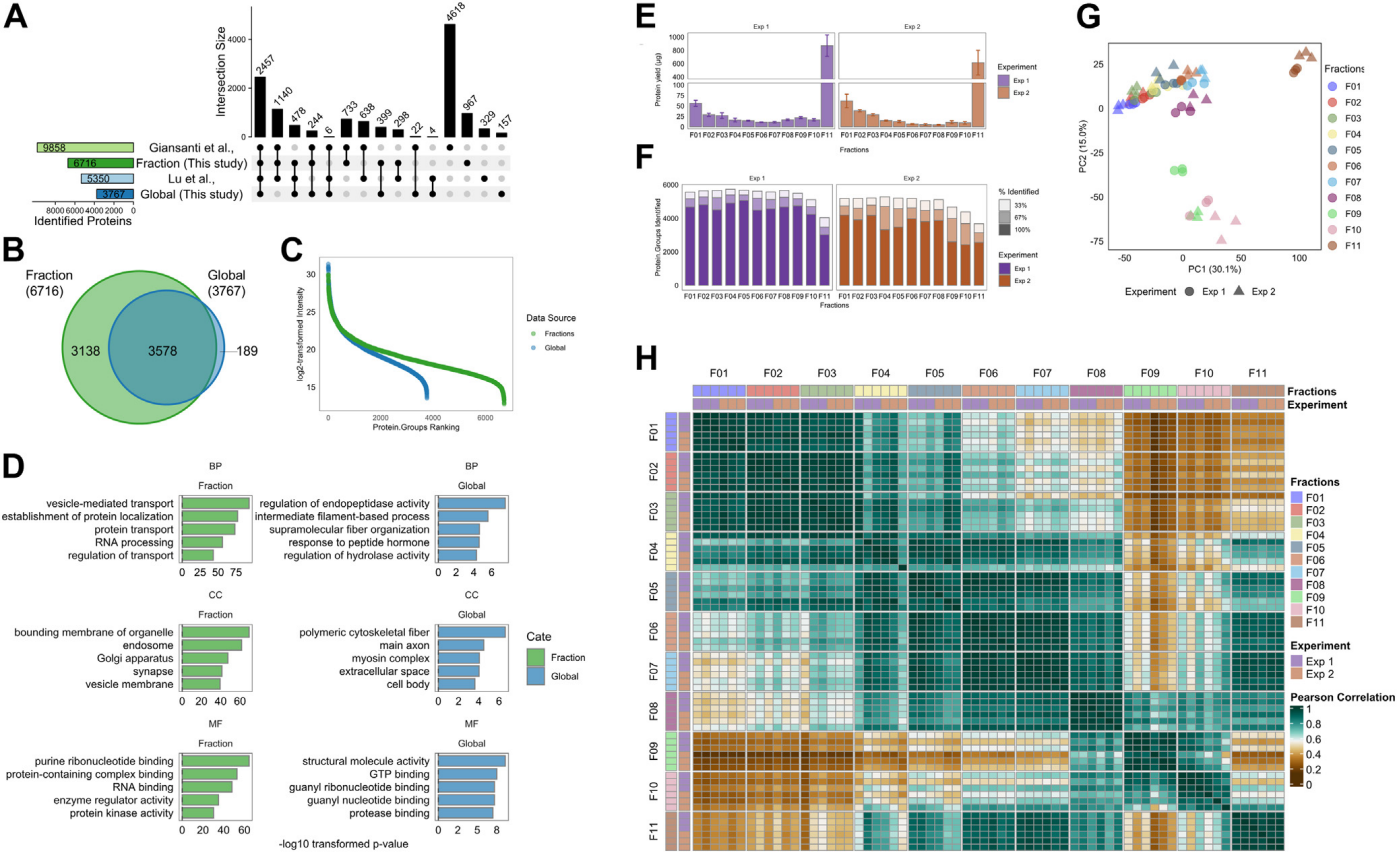
**Reproducible subcellular fractionation proteomics workflow of mouse heart.***A*, upset plot comparison of identified proteins from single-shot global mouse heart proteome (global), subcellular fractionated mouse heart proteome (fraction), and previously published mouse heart proteome data. *B*, Venn diagram analysis of identified proteins. *C*, dynamic range of log2-transformed protein group intensities. *D*, bar chart of significantly enriched gene ontology (GO) terms of uniquely identified proteins from each data set. *E*, bar plots of protein yield from distinct subcellular fractions (F01-F11) of mouse heart tissue. Two independent experiments (Exp1, Exp2) were commenced with three biological replicates (error bar: ± sd). *F*, stacked bar plots of identified protein groups across independent experiments with corresponding identification ratio (% of three biological replicates) of distinct subcellular fractions. *G*, principal component analysis (PCA) of log2-transformed proteome data of subcellular fractions (F01-F11). *H*, Pearson correlation matrix for subcellular fraction proteome.

We applied our pipeline to two independent experiment sets of biological triplicates of mouse heart extracts (Fig. 2). A small portion of the heart extract was used for single-shot (Global) DIA-MS proteome analysis, followed by subsequent differential centrifugation–based fractionation of 11 subcellular fractions (fraction) (Fig. 2, *A*–*D*). Collectively, we identified 6716 protein groups from mouse heart subcellular proteome (Fraction), a significant increase in depth compared to 3767 proteins from global heart proteome (Fig. 2, *A* and *B*, Supplemental Table S1). Our subcellular proteome data supports the coverage of previous mouse heart proteome employing extensive peptide fractionation (62, 63) (Fig. 2*A*) and reveals a significantly increased dynamic range in comparison to the single-shot proteome data (Fig. 2*C*, Supplemental Table S2). Proteins uniquely identified from our fractionation approach were enriched for GO terms such as “endosome” and “Golgi Apparatus” (GO cellular component), and “vesicle-mediated transport” and “RNA processing” (GO biological processes), highlighting improved resolution of subcellular proteins following subcellular fractionation (Fig. 2*D*, Supplemental Table S3).

With respect to reproducibility, we obtained similar protein yields from each fraction across two independent preparations (Fig. 2*E*, Supplemental Table S4). We report 5134 commonly identified protein groups from both experiments. On average, around 5100 proteins were quantified in each fraction and more than 90% quantified in at least two out of three replicates with maxLFQ algorithm (Fig. 2*F*, Supplemental Table S2).

For data processing, we have conducted VSN following removal of batch-effect using limma package and applied imputeLCMD for missing values at random with neighborhood-based K-nearest neighbors imputation and missing values not at random with left-censored QRILC method. We examined the coefficient of variation of protein group intensities for each fraction (Supplemental Fig. S1*A*, Supplemental Table S5) to assess the quantitative variability for label-free DIA-MS data (median CV = 17.8%, Supplemental Table S5). We also report a median Pearson correlation at 0.71 comparing the intensity profiles of 5134 protein groups of individual replicates (Exp1: n = 3, Exp2 n = 3, Supplemental Fig. S1*B*, Supplemental Table S6). PCA revealed that proteomes of the same fraction from different experiments cluster in proximity (Fig. 2*G*). This was further supported by Pearson correlation matrix analysis which demonstrates reproducible measurements between replicates for each fraction (Pearson correlation coefficient: 0.725–0.996, Fig. 2*H*, Supplemental Table S7). This analysis also revealed major clusters in our dataset between fractions 1 to 3, fractions 4 to 7, fraction 8, fractions 9 to 10, and fraction 11. To evaluate the influence of data-processing pipeline on our data, we first visualized the heatmap of 66 samples based on log2-transformed intensity prior to data imputation (Supplemental Fig. S1*C*) and plotted nonlinear iterative partial least squares-PCA at different stages of data processing (Supplemental Fig. S1, *D*–*F*). From these analyses, we conclude that our experiment 1 and 2 datasets are highly comparable, with the applied data processing pipeline resolving interexperimental batch effect through normalization while preserving the overall subcellular proteome data structure. We demonstrate that our workflow can reproducibly quantify the subcellular proteome of the mouse heart in depth.

### Subcellular Fractionation Resolution to Resolve the Heart Subcellular Compartments

**Fig. 3:**
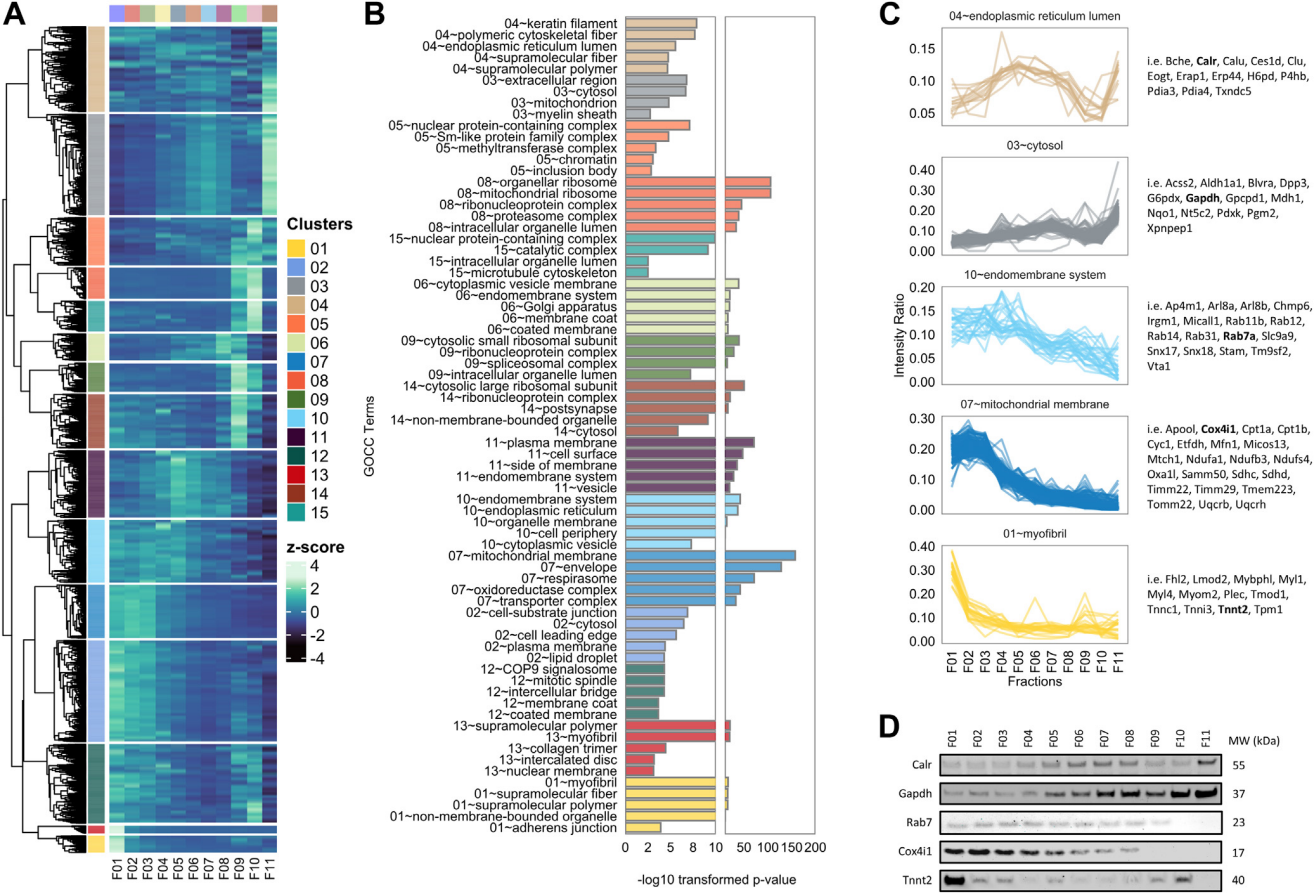
**Sequential fractionation resolves subcellular proteome of the mouse heart.***A*, heatmap of z-score normalized protein intensities across distinct subcellular fractions using sequential centrifugation of mouse heart combined for each experiment. Fifteen clusters were distinguished based on k-means clustering of the average profile of 5134 protein groups. *B*, top five significantly enriched gene ontology (cellular compartment) terms of proteins identified from each cluster (Benjamini–Hochberg adjusted *p*-value <0.05). *C*, profile intensity plots of scaled average protein intensity across subcellular fractions from each significantly enriched GOCC terms shown. *D*, Western blot validation of selected marker proteins and their expression (abundance) distribution across isolated fractions.

To delineate protein features enriched between fractions, we applied unsupervised hierarchical clustering (partition-based k-means clustering) which revealed 15 distinctive protein clusters (Fig 3*A*, Supplemental Fig. S1, *A* and *B*, Supplemental Table S8). We analyzed the protein group composition of each cluster pattern via GO annotation enrichment analysis for cellular component (Benjamini–Hochberg adjusted *p*-value<0.05, Fig. 3, *B* and *C*, Supplemental Tables S8 and S9). Interestingly, we observed that many structural or nuclear proteins are copurified and enriched in fraction 1 (F01), including myofibril, collagen trimer, or nuclear membrane (Fig. 3*B*, Supplemental Tables S8 and S9), similar to previous studies (64, 65). On the other hand, many proteins involved in mitochondria- and cell-junction–associated plasma membrane are copurified and enriched in fraction 2 (F02). Further, we resolve proteins associated with endomembrane system including endoplasmic reticulum, cytoplasmic vesicle, Golgi apparatus, and plasma membrane from fractions F04 to 08. Protein complexes such as nuclear protein–containing complex, organellar ribosomes, and proteasome complex are enriched in high centrifugation force fractions (F09–10). Lastly, cytosolic and select proteins of endoplasmic reticulum and mitochondrion were identified in the supernatant post serial centrifugation (F11).

As orthogonal validation, we performed Western blot analysis of 11 subcellular fractions using known protein markers for cytosol (Gapdh), ER lumen (Calr), late endosome (Rab7a), mitochondria (Cox4i1), and myofibrils (Tnnt2) as described (30, 66) (Fig. 3*D*, Supplemental Fig. S2, *B*–*F*). We demonstrate enrichment of select organelle protein markers and their distribution and supports our fractionation resolution combined with MS-based profiling for distinct cell compartments (Fig. 3, *C* and *D*).

A pressing challenge in processing frozen samples is preserving organelle integrity (44, 67). In this regard, we did identify proteins residing in the same organelle but revealed differential subcellular fractionation profile. For example, ER proteins are enriched in cluster 10 (i.e., Ryr2, Trdn, Ddost, Emc8), whereas a subpopulation of ER lumen proteins (i.e., Calr, Calu, H6pd, Txndc5) are enriched in cluster 4 (Fig. 3, *B* and *C*). Similarly, we further identify mitochondrial membrane proteins in cluster 7 (i.e., Apool, Cox4i1, Cpt1a, Cyc1) and other mitochondrion proteins in cluster 3 (i.e., Ckmt1, Sod2, Oat, Acadm). Despite the limitation of preserving of organelle integrity from heart tissue, our data demonstrates consistent fractional profiles of these suborganelle protein features across replicates (Supplemental Fig. S2*G*).

### ML-Driven Classification of Heart Subcellular Proteome

Next, we leverage different supervised ML algorithms on quantitative fractionation profiles to map mouse heart proteins to their respective subcellular niches. For this, we initially curated our heart-centric protein marker list, built and refined our model for subcellular protein classification, tested the performance of individual models, and constructed a high-confidence subcellular protein annotation map.

**Fig. 4:**
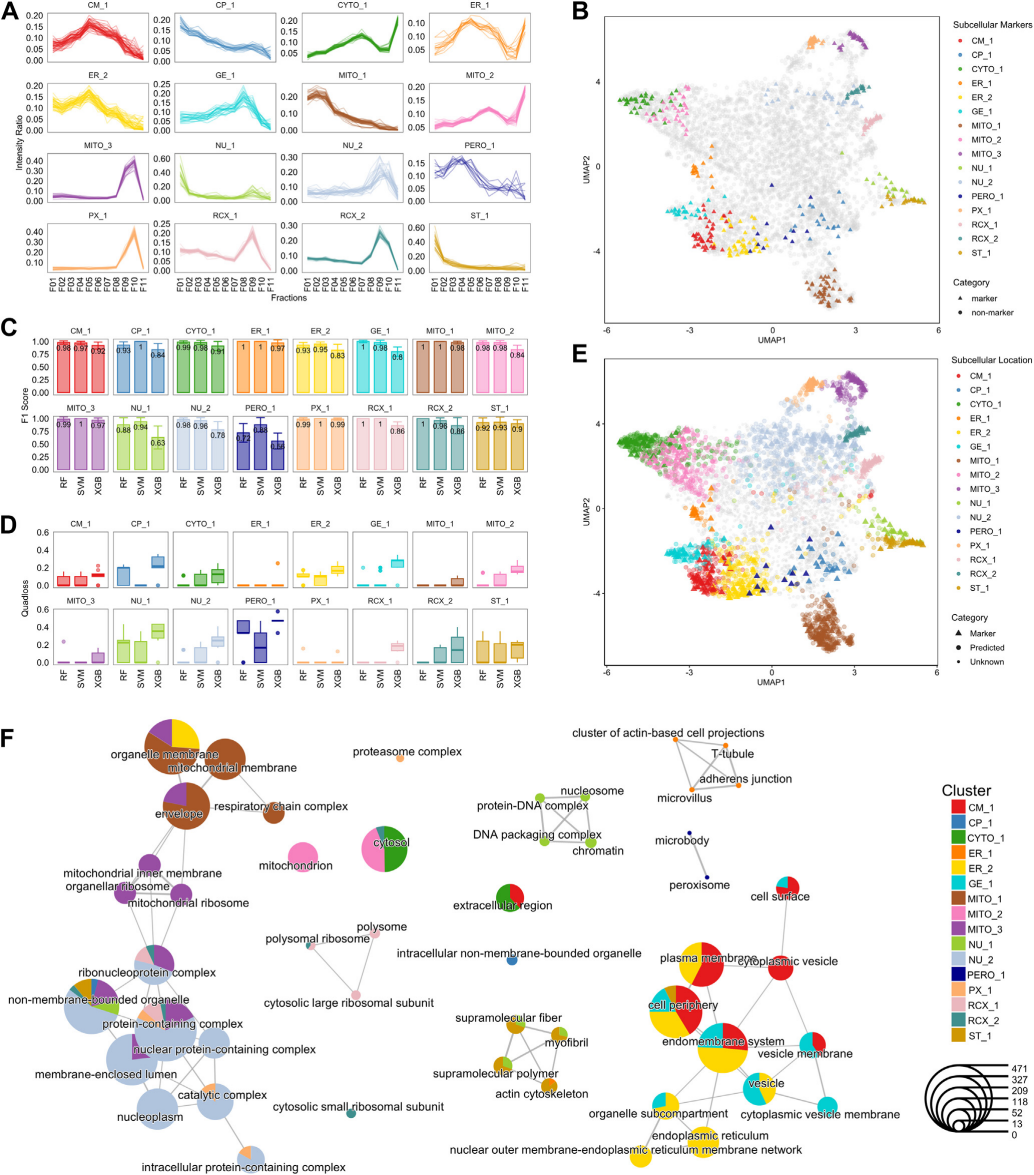
**Supervised machine learning–assisted protein subcellular localization annotation.***A*, profile plots of 16 different subcellular niches (CM_1: cell membrane, CP_1: cell periphery, CYTO_1: cytosol, ER_1: endo-/sarcoplasmic reticulum lumen, ER_2: endo-/sarcoplasmic reticulum membrane, GE_1: Golgi apparatus/endo-lysosome, MITO_1: mitochondrial membrane, MITO_2: mitochondrial matrix, MITO_3: mitochondrial ribosomes, NU_1: chromatin associated nucleus, NU_2: nucleoplasm, PERO_1: peroxisome, PX_1: proteasome, RCX_1: large ribosomes, RCX_2: small ribosomes, ST_1: structural proteins) based on the means of 0 to 1 scaled intensity ratios of curated organelle protein markers. *B*, 2D UMAP plot showing the spatial dispersion of marker proteins of the 16 subcellular niches. *C*, bar chart of mean F1 scores of each organelle proteome prediction from SVM, RF, and XGBoost output (error bar: ± sem). *D*, boxplot of median and quantiles of quadratic losses of each organelle proteome prediction from SVM, RF, and XGBoost output. *E*, 2D UMAP plots of predicted protein localizations based on comatched features from SVM and RF with FDR <0.05. *F*, enrichment map of top GO cellular compartment terms based on functional enrichment analysis of predicted protein groups from 16 subcellular niches (q-value <0.05).

We first manually curated high confidence proteins in different organelles from various databases including GO (52), Mouse Genome Informatics (51), UniProt (50), and Human Protein Atlas (49). We integrated previously published data with in-depth subcellular protein assignments (29, 47, 48) to collate a subcellular heart-centric resource (Supplemental Table S10). This resulted in 450 protein markers from different subcellular niches including cell membrane (CM), cell periphery/cell junction (CP), cytosol (CYTO), ER/sarcoplasmic reticulum, Golgi apparatus/endo-lysosome (GE), mitochondria (MITO), nucleus (NU), peroxisome (PERO), proteasome (PX), ribosome (RCX), and structural proteins (ST) (Fig. 4*A*). For specific subcellular niches, we further classified proteins annotated from ER (ER_1: endo-/sarcoplasmic reticulum lumen, ER_2: endo-/sarcoplasmic reticulum membrane), MITO (MITO_1: mitochondrial membrane, MITO_2: mitochondrial matrix, MITO_3: mitochondrial ribosomes), NU (NU_1: chromatin associated nucleus, NU_2: nucleoplasm), and RCX (RCX_1: large ribosomes, RCX_2: small ribosomes) into distinct subcategories (Fig. 4*A*). UMAP supports the spatial resolution of these 16 subcellular categories (Fig. 4, *A* and *B*). We assessed the subcellular resolution of each subcellular category via Qsep analysis to compare the average Euclidean distance of intrasubcellular and intersubcellular categories (Supplemental Fig. S3*A*). The Qsep analysis verified the considerable separations (average Qsep distance = 3.00) among different subcellular niches of the heart subcellular proteome (Supplemental Table S11).

Next, we used the fractionation profile of these 450 protein markers to build different ML subcellular classification models. For this, we incorporated different algorithms including SVM, RF, XGB from pRoloc and pRolocExtra packages (45, 61) for supervised ML. Hyperplane-based SVM and decision tree–based RF from pRoloc have been applied to different subcellular proteomics studies with demonstrated performance (27, 29, 32, 68). We included decision tree–based algorithm XGB due to its advancement in avoiding model overfitting and applications in proteomics data annotation (69, 70). We fine-tuned our model by performing parameter optimization of these algorithms with 100 iterations and 5-fold cross-validation, selecting the optimal parameters for each based on the macro-F1 score which assess the harmonic mean of precision and recall of different parameter pairs (indicative of model performance) (Supplemental Fig. S3, *B*–*D*, Supplemental Tables S12 and S13). With optimized parameters for SVM, RF, and XGB, we then evaluated the predictive performance of each algorithm. The average F1 scores of SVM (mean F1 = 0.97) and RF (mean F1 = 0.96) are consistently higher than XGBoost (mean F1 = 0.85), indicating a higher precision and recall of SVM and RF in comparison to XGB in classifying different subcellular features (Fig. 4*C*, Supplemental Table S14). The lower average quadratic losses/mean square error of SVM (0.05) and RF (0.06) also confirmed a higher accuracy than XGB (0.16) (Fig. 4*D*, Supplemental Table S15).

We next tested the performance of SVM-, RF-, and XGB-based models to the entire subcellular proteome (5134 protein groups) for subcellular classification. Since our models have shown different level of performance in predicting proteins of different subcellular categories (Fig. 4, *C* and *D*), we employed organelle-specific threshold (high confidence FDR <0.05) for individual subcellular categories to account for the predictive performance of each subcellular niche. For each algorithm, we incorporated a rank-based cutoff where predicted proteins of each category were ordered based on their probability score (Supplemental Tables S16 and S17). We found that SVM, RF, and XGB have categorized 2674, 2780, and 2158 proteins (FDR <0.05), respectively (Supplemental Fig. S3, *E*–*G*, Supplemental Table S18). Additionally, we evaluated these independent ML algorithms in predicted overlapping protein subcellular classifications, highlighting reliability and confidence in our modeling. In this regards, we found SVM and RF provide the highest agreement in prediction and confident assignment of subcellular niches for the mouse heart proteome (Supplemental Fig. S3*H*). Therefore, to construct a heart subcellular proteome with high stringency, we only incorporated proteins predicted with high confidence based on both SVM and RF. This resulted in the annotation of 2083 proteins to their 16 subcellular categories (Fig. 4*E*, Supplemental Table S18). Specifically, these include CM_1: 144 proteins, CP_1: 17, CYTO_1: 280, ER_1: 10, ER_2: 197, GE_1: 96, MITO_1: 302, MITO_2: 240, MITO_3: 91, NU_1: 32, NU_2: 513, PERO_1: 2, PX_1: 33, RCX_1: 61, RCX_2: 16, ST_1: 49. UMAP analysis reveals the spatial segregation of protein components for each of these subcellular categories. Expectantly, we show the spatial arrangements of the predicted protein components highly align with known corresponding protein markers for each niche (Fig. 4*E*).

Next, we investigated the enrichment of cell compartment localization relative to each category based on the 2083 predicted subcellular proteins. Here, we employed Clusterprofiler to investigate protein-based subcellular localization, indicating a high degree in alignment (CM_1, ER_2, GE_1, MITO_1, MITO_3, NU_2, PERO_1, PX_1, RCX_1, RCX_2) with their predictive subcellular annotations (Fig. 4*F*, Supplemental Table S19). We also found that shared GO enrichment terms are enriched between CYTO_1 and MITO_2; and NU_1 and ST_1, which might be due to their highly similar fractionation profiles, and low Qsep distances between these groups (Supplemental Fig. S3*A*, Supplemental Tables S11 and S19). For instance, the predicted proteins from NU_1 and ST_1 category have shared GO enrichment terms in “myofibril.” In addition, we report protein categories with slightly ambiguous subcellular protein prediction outcomes including CP_1 and ER_1 (Fig. 4*F*). CP_1 category is not enriched with highly significant localization terms; however, the functional annotations of CP_1 proteins revealed their close associations with endosomal pathways (i.e. Ehd4, Spart, Rufy1), cell junctions (i.e. Pdcd6ip, Tln1, Tln2,), and glycoprotein complex (i.e. Flna, Snta1). ER_1 category is enriched with terms such as “sarcoplasmic reticulum” and “T-tubule,” a specialized plasma membrane domain that in proximity with sarcoplasmic reticulum (Supplemental Table S19). Highlighting the similar fractionation profiles of proteins residing in these cellular compartments may require further investigation to delineate their localization using orthogonal fractionation or higher resolution approaches.

Here, we also report the suborganelle niches of mitochondria (MITO_1, MITO_2, MITO_3), likely associated with organelle disruption from highly structured heart tissue during sample homogenization. Encouragingly, our data reveal distinct profile patterns and clusters corresponding to the mitochondrial membrane, matrix, and ribosomes, distinguishable from other subcellular niches. GO-based analysis also confirms the alignment of predicted proteins with their anticipated annotated functions (Supplemental Table S19). Further, comparative analyses with detergent-based subcellular fractionation on highly structured snap-frozen mouse skeletal muscle revealed similar organelle proteome patterning (44). Both datasets were closely aligned, revealing distinct protein profile patterns between mitochondrial electron transport chain proteins (i.e. Cox5a, Ndufa2, Uqcrfs1), matrix proteins (i.e. Acot2, Aldh2, Etfa), and ribosomal proteins (i.e. Mrpl20, Mrps2, Mterf2). Therefore, we demonstrate the construction of a high confidence mouse heart subcellular proteome (2533 proteins; 450 marker proteins with 2083 predicted proteins).

### Validation of the Cardiac Subcellular Proteome

With a highly confident subcellular protein map of mouse heart in place, we next leveraged published studies on heart-centric organelle enrichment (24, 25, 26, 64, 71, 72, 73, 74, 75, 76, 77, 78, 79) (Supplemental Table S20). First, to analyze our subcellular proteomics data at a system level, we have compared confidently annotated subcellular proteins from our proteomics data (2533 proteins) with those with consistent subcellular assignments from Currie *et al*., (64). From these two datasets, 887 proteins are co-identified, of which 661 proteins reported with matching subcellular locations (Supplemental Table S21, Supplemental Fig. S4*A*). Overall, proteins residing in large organellar membranes (i.e. MITO_1, ER_2), protein complexes (i.e., MITO_3, PX_1, RCX_1, PCX_2), and cytosolic region (i.e. CYTO_1) have demonstrated higher proportion of matching subcellular locations. The difference or unmatching annotations may be due to 1) cellular heterogenicity of mouse tissue, 2) structurally different iPSC-derived cardiomyocyte versus heart tissue, 3) taxonomically different proteins with differential subcellular location, as well as 4) inaccuracy in protein subcellular organelle assignments. Despite these differences, the list of high-confident subcellular-assigned proteins provides a draft map of subcellular proteome from *in vivo* heart tissue.

**Fig. 5:**
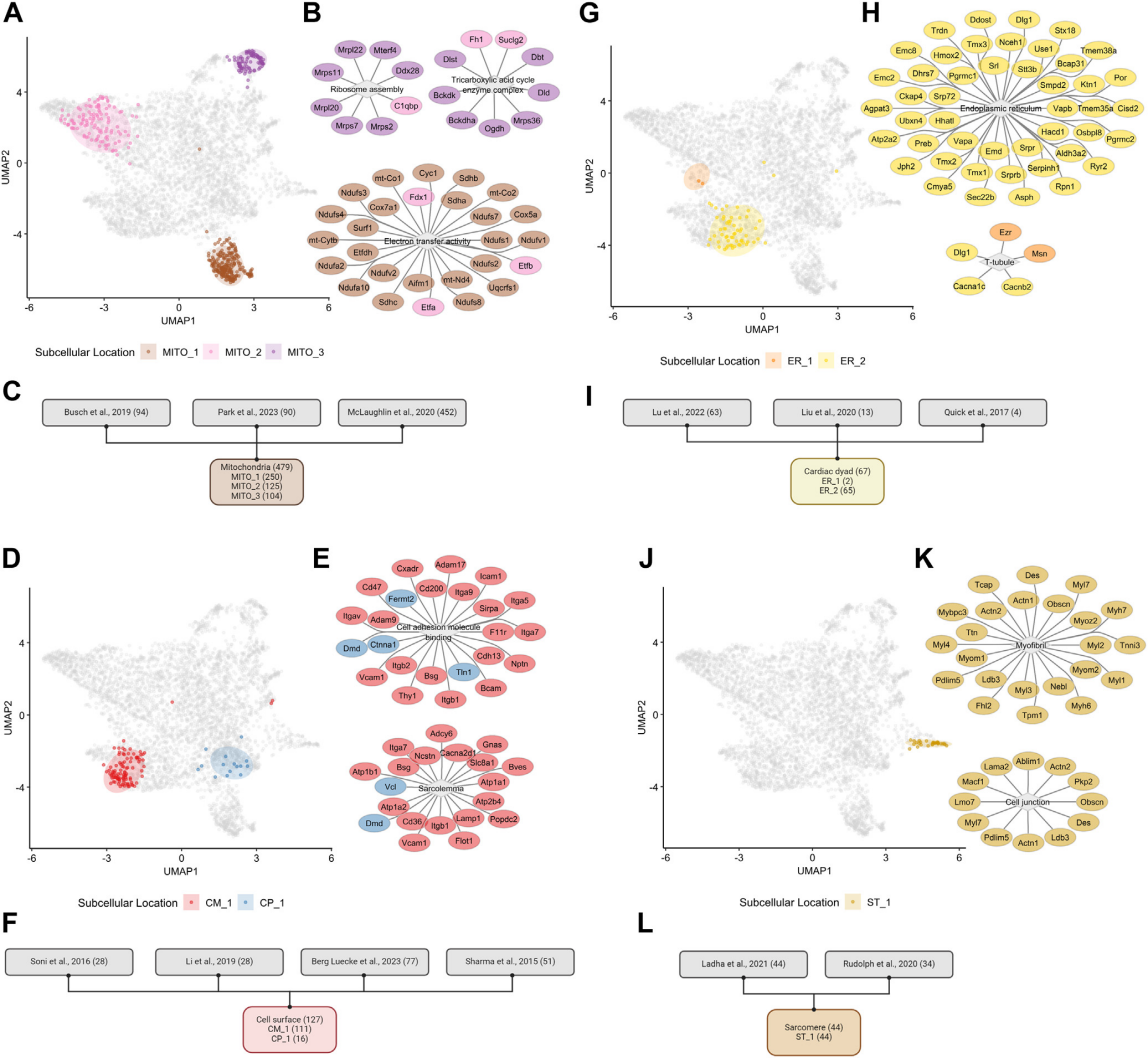
**Aligning mouse heart subcellular proteome with subcellular proteomics datasets of cardiovascular system.** Two dimensional UMAP of overlapping protein entities between our study and other subcellular proteomics studies, highlighting the spatial arrangement of mitochondria (*A*), cell surface (*D*), cardiac dyad (*G*), sarcomere(*J*). The shaded areas represent the ellipse of marked subcellular categories (confidence level of multivariate t-distribution = 0.85). Overlapping proteins are highlighted in UMAP plots. Selected key KEGG/Reactome/GO-enriched protein networks of co-identified proteins from this study and previously reported proteomic data from mitochondria (*B*), cell surface (*E*), cardiac dyad (*H*), sarcomere (*K*) are visualized via Cytoscape. The flow charts represent the numbers of overlapping proteins identified from previous publications focused on mitochondria (*C*), cell surface (*F*), cardiac dyad (*I*), sarcomere (*L*).

Next, we analyzed proteins from selected subcellular niches including mitochondria, cell surface, cardiac dyad, and sarcomere comparing previously published data with our subcellular proteome data (Supplemental Tables S20 and S21). We first compared our heart mitochondria proteome with mitochondrial-specific proteome data using co-immunoprecipitation (72), proximity-labeling (71), and differential centrifugation (24) approaches. Overall, 479/633 (76%) mitochondrial proteins were co-identified in mouse heart mitochondria proteome (Fig. 5, *A*–*C*). Through functional enrichment analysis, these proteins are comprised of mitochondrial “ribosome assembly (GO:0042255, FDR = 0.0082),” “tricarboxylic acid cycle enzyme complex” (GO:0045239, FDR = 3.97E-08), “electron transfer activity” (GO:0009055, FDR = 2.21E-22), and proteins networks associated with “response to oxidative stress” (GO:0006979, FDR = 5.85E-05) and “fatty acid metabolism (MMU-8978868, FDR = 1.40E-05) (Fig 4*B*, Supplemental Fig. S4*B*, Supplemental Tables S22).

In heart tissue, specifically cardiomyocyte cell surface consistent of specialized membrane domains such as t-tubules and intercalated discs (ICD), which are crucial for cell-cell communication and signal conduction. Here, we co-identified 127/224 (57%) cardiac cell membrane proteins from studies investigating cardiac cell surface network or targeted studies on ICD proteome (76, 77, 78, 79) (Fig. 5, *C* and *D*, Supplemental Table S21). We report significantly enriched terms in “sarcolemma” (GO:0042383, FDR = 5.60E-18), “cell adhesion molecule binding” (GO:0050839, FDR = 1.37E-16), and “plasma membrane bounded cell projection” (GO:0120025, 1.58E-19), which are all highly relevant to cell surface function (Fig 4*E*, Supplemental Fig. S4*C*, Supplemental Table S22). Additionally, we have confirmed the presence of multiple ICD proteins (i.e., Atp1a1, Atp1a2, Cxadr, Slc8a1) from our data.

Cardiac dyad is a highly regulated subcellular niche connecting t-tubule and junctional sarcoplasmic reticulum. Cardiac dyad facilitates the excitation–contraction via controlling local calcium ions (25). We co-identified 67 ER-associated proteins with proteomic studies targeting the cardiac dyad (25, 73, 74) (Fig. 5, *E* and *F*, Supplemental Table S21). The functional enrichment analysis further confirms their subcellular annotations with “endoplasmic reticulum” (GO:0005783, FDR = 7.37E-32) and “t-tubule” (GO:0030315, FDR = 2.00E-04) (Fig 4*H*, Supplemental Fig. S4*D*, Supplemental Table S22).

**Fig. 6:**
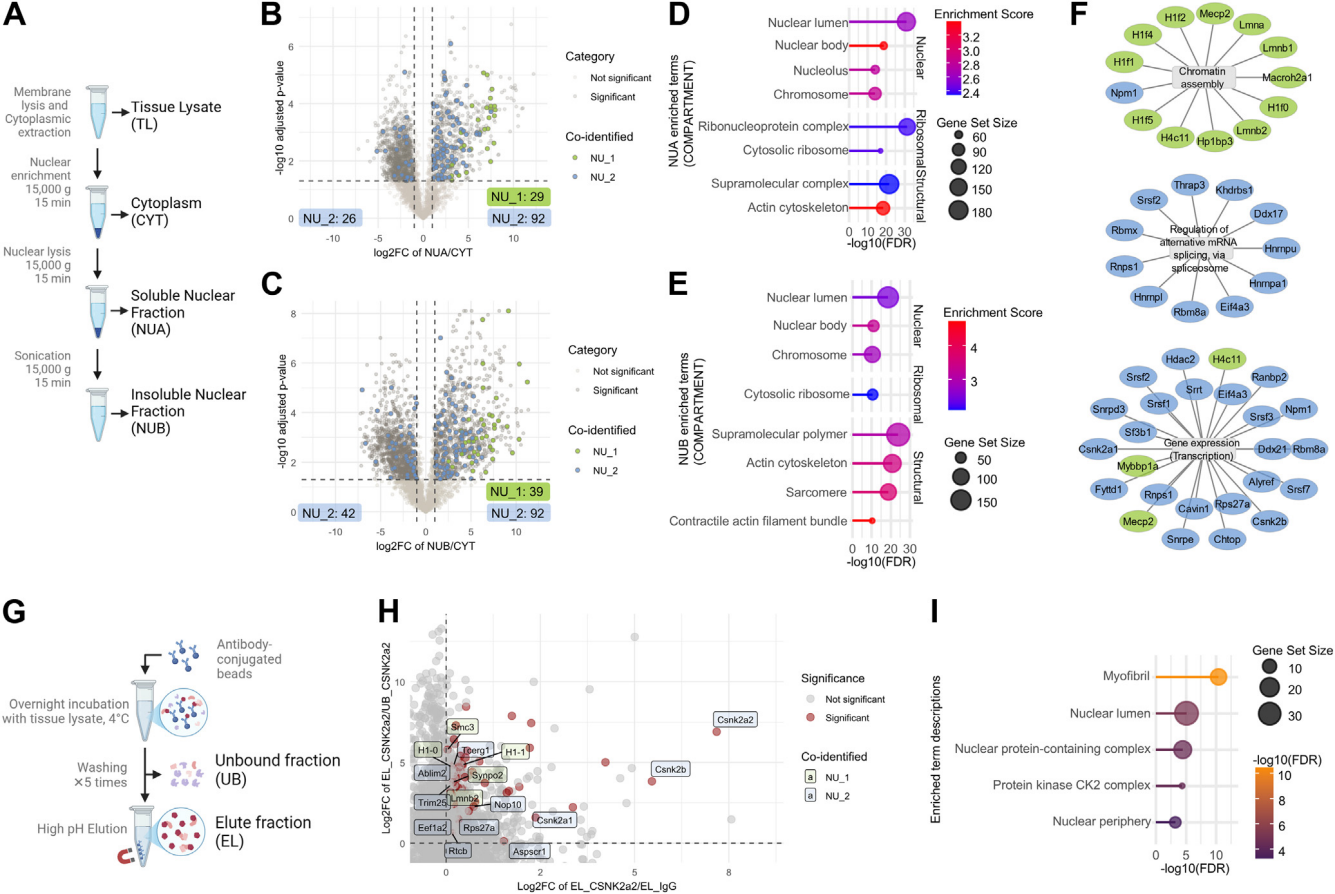
**Experimental validation of cardiac nuclear proteome.***A*, simplified experimental workflow of targeted nuclear enrichment from mouse heart, nuclear fractions (NUA, NUB), and cytoplasmic fraction (CYT). Volcano plots generated based on student’s tests of log2-transformed protein group intensities of NUA versus CYT (*B*) and NUB versus CYT (*C*). Significant differentially abundant protein groups (BH-adjusted *p*-value <0.05, |log2FC| > 1) are highlighted in *dark gray*. Co-identified protein groups from NU_1 and NU_2 subcellular proteome data are highlighted in *green* and *blue*, respectively. Lollipop charts highlight the significantly enriched GOCC terms based on functional enrichment analysis of significantly different protein groups from NUA versus CYT (*D*) and NUA versus CYT (*E*). Selected GOCC protein clusters with highlighted co-identified protein (gene names) from NU_1 and NU_2 subcellular proteome data (*F*). *G*, simplified experimental workflow of co-IP experiment. *H*, from co-IP experiment, scatter plot of log2-FC of protein intensities from Csnk2a2 elute fraction (EL_Csnk2a2) versus Csnk2a2 unbound fractions (UB_ Csnk2a2) on y-axis and Csnk2a2 elute fraction (EL_Csnk2a2) versus IgG elute fractions (EL_IgG) on x-axis. Csnk2a2-associated proteins (BH-adjusted *p*-value <0.05, in either EL_Csnk2a2 versus UB Csnk2a2 or EL_Csnk2a2 versus EL_IgG comparisons and log2-FC >1 in both comparisons) are highlighted in *dark red*. Co-identified protein groups from NU_1 and NU_2 subcellular proteome data are labeled in *green* and *blue*, respectively. *I*, the lollipop charts highlight the significantly enriched GOCC terms of Csnk2a2 strongly associated proteins.

We further integrated proteome analyses of the sarcomere, a functional unit of cardiac contraction. We co-identified 44/69 (64%) proteins with cardiac sarcomeric proteome previously reported using proximity labeling (26, 75) (Fig. 5, *G* and *H*, Supplemental Table S21) with enriched terms in “myofibril” (GO:0030016, FDR = 1.03E-34) and “cell junction” (GO:0030054, FDR = 1.80E-03) (Fig 4*K*, Supplemental Fig. S4*E*, Supplemental Table S22). Here, our data demonstrated resourceful knowledge when combining with other cardiac-specific subcellular proteomics studies. Further, to validate the predicted sarcomere proteome, we performed Co-IP of cardiac troponin T (Tnnt2) to investigate the immediate binding partners of this sarcomeric protein (Fig. 6*G*). Using differential expression, we report myofibril proteins (i.e. Tnni3, Myom1, Myom2, Tpm1) and cell junction proteins (i.e. Macf1, Actn1) significantly associated with Tnnt2 in comparison to unbound proteins from Tnnt2 Co-IP or associated with nonspecific IgG (FDR <0.05, log2FC > 1) (Supplemental Fig. S4*F*, Supplemental Table S23).

### Validation of Nuclear Protein Networks in the Cardiac Subcellular Proteome

Cardiac nuclei are crucial for regulating different cardiac phenotypes via the changes of chromatin arrangements (80, 81, 82) and the regulations of transcription factors (83, 84, 85); yet it remains relatively understudied in proteomics research in comparison to other cardiac organelles. Here, we validated the nuclear category (NU_1 and NU_2) of subcellular proteome map via targeted enrichment using a stepwise detergent lysis approach to derive cardiac nuclei (NUA, NUB) and cytoplasm (CYT) from mouse heart (Fig. 6*A*, Supplemental Table S24). Based on their differential expression (one-way ANOVA, FDR < 0.05), we report the enrichment of “nucleolus” and “ribonucleoprotein complex” in NUA and “nuclear envelope” and “chromosome” in NUB (Supplemental Fig. S4, *G* and *H*, Supplemental Table S25 and S26). Further, we show that NUA and NUB proteome are highly dissimilar from CYT, with >50% of proteome (NUA vs CYT: 1766 out of 3438; NUB vs CYT: 1951 out of 3438) significantly different (FDR < 0.05, |log2FC| >1) (Fig. 6, *B* and *C*, Supplemental Table S24). To reveal molecular composition of these different protein networks in NUA/B, GO-based functional enrichment analysis revealed enrichment of structural proteins including “Actin cytoskeleton” and “Sarcomere” proteins; a challenge of isolating nuclei from cardiomyocytes and highly fibrous tissue (64, 86) (Fig. 6, *D* and *E*, Supplemental Table S29). Importantly, we observed significantly enriched nuclear (i.e. “Nuclear lumen”, “Chromosome”) and ribonucleoproteins (i.e. “Ribonucleoprotein complex”, “Cytosolic ribosome”) in NUA and NUB groups (Fig. 6, *D* and *E*, Supplemental Table S29).

Next, we mapped proteins identified from NU_1 and NU_2 to this nuclear-enriched dataset from mouse heart (Supplemental Table S24). As demonstrated in both volcano plot, co-identified NU_1 and NU_2 group proteins are predominantly found in NUA (NU_1: 29, NU_2: 92) and NUB (NU_1: 39, NU_2: 92) groups versus CYT fraction (Fig 6, *B* and *C*, Supplemental Fig. S4*I*). The heatmap further reveal that 79.4% of co-identified NU_1 and NU_2 proteins are significantly enriched in either or both NUA and NUB groups versus CYT (Supplemental Fig. S4*I*, Supplemental Table S24). Through functional enrichment analysis, these co-identified and nuclear-enriched NU_1 and NU_2 protein groups are strongly associated canonical nuclear functions, such as chromatin assembly (Macroh2a1, Lmna, Lmnb1, Mecp2, Hp1bp3, H4c1), spliceosome-associated mRNA splicing (Hnrnpa1, Thrap3, Srsf2, Rbmx), and gene expression (Hdac2, Mecp2, Mybbp1a, Csnk2a1) (Fig. 6*F*, Supplemental Table S28).

To further verify the nuclear portions of our subcellular proteome map, we have conduced Co-IP MS to identify the protein partners of predicted NU_2 category protein casein kinase II subunit alpha (Csnk2a2) (Fig. 6*G*). We have applied stringent filtering of protein groups, which are strongly associated with Csnk2a2 Co-IP (bound) relative to either unbound fraction or nonspecific IgG (FDR <0.05, log2FC > 1) (Fig 6*H*, Supplemental Fig. S4*J*, Supplemental Table S23). In mapping these Csnk2a2 Co-IP (bound) protein network to our subcellular protein UMAP, we reveal a high proportion of this protein network associating with either NU_1 (chromatin- and nuclear-envelope–associated proteins) or NU_2 (nucleoplasm-associated proteins) clusters (Supplemental Fig. S4*K*). The functional enrichment analysis of these Csnk2a2-associated protein network reveals “myofibril” and “nuclear lumen” signatures, aligning with our targeted nuclear-enrichment proteomic data (Fig. 6*I*, Supplemental Table S29). Additionally, we also identified all components of “Protein kinase CK2 complex” (Csnk2a1, Csnk2a2, Csnk2b) from this Co-IP experiment (Fig. 6*H*). From the list of Csnk2a2 significantly associated proteins, we co-identified 16 proteins from NU_1 and NU_2 categories (Fig. 6*H*), of which 12 protein groups are also identified in targeted nuclear-enrichment proteomic data and 11 protein (Ablim2, Csnk2a1, Csnk2b, Eef1a2, H1-0, H1-1, Lmnb2, Rps27a, Rtcb, Smc3, Synpo2) intensities significantly higher in nuclear fractions.

## Discussion

Subcellular localization of cardiac cell organelles is crucial for heart function. Profiling subcellular niche provides crucial insights in understanding cardiac physiopathology. Here, we provide a reproducible approach to fractionate and define the subcellular proteome of mouse heart in a holistic manner. Our study utilizes differential centrifugation–based subcellular fractionation, label-free DIA MS–based proteomic profiling and evaluation of different ML approaches to provide a comprehensive map of spatial regulation of cardiac protein networks. From 5134 protein groups with quantitative fractionation profiles, we applied SVM- and RF-based ML approaches and annotated 2533 (49.3%) protein groups to 16 subcellular niches with high confidence. We further confirmed protein subcellular locations with GO-based functional enrichment analysis and revealed key protein components of four cardiac-specific subcellular niches (mitochondria, cell surface, myofibril, cardiac dyad) as well as targeted enrichment of nucleus from the heart combined with Co-IP and proteomic profiling, validating our proteomics and ML-based approach to decipher subcellular protein features of mouse heart.

In the past, spatial subcellular proteomic studies have been investigated at cellular level using cell lines, primary cell cultures, and single cell organisms (32, 34, 87, 88, 89, 90). Although the studies of holistic subcellular protein distribution at tissue level remain limited, such studies are crucial to capture the influence of intercellular communication of different cell populations within their local environment on subcellular protein distributions. Thus, studying the dynamics of protein subcellular distribution at a tissue level can provide biologically relevant molecular insights into physio- and patho-logical remodeling. For instance, Kandigian *et al*. profiled subcellular proteome of human brain tissue from patients with Alzheimer’s disease, revealing a nuclear-to-cytoplasmic shift of proteins crucial to the onset of disease hallmarks when dysregulated (67). Further, Martinez-Val *et al*. highlighted an increased proportion in the ratio of nuclear:cytoplasmic ribosomal proteins in mouse skeletal muscle following mechanical stress, indicative of ribotoxic stress response at a subcellular level (44).

Resolving subcellular niches of cardiac tissue presents with numerous technical and analytical challenges (27, 44, 67), including limited sample availability, tissue homogenization, and proteome coverage. To meet these challenges, we optimized our experimental procedures to conduct reproducible and in-depth subcellular proteome map of mouse heart with just 40 mg of tissue material with 10 h of MS run time of 11 subcellular fractions. For sample processing, we commenced rapid (<10 min) tissue dissociation and lysis at 4 °C to maximize the preservation of intact organelles with minimal cross contamination. Similar extraction approaches have been employed in highly structured tissues including heart (91) and skeletal muscle (44). Due to various challenges in source and access to nonfrozen tissue samples, including the heart, we optimized our workflow using frozen tissue to extend the application of this pipeline to many archived samples from various resources and biobanks.

To improve our fractionation workflow, we also incorporated an additional centrifugation stage at 600*g*, previously reported to enrich for myofibril and extracellular proteins from heart and skeletal tissue (92, 93). Further, we employed label-free DIA-MS for proteomic profiling. Although label-based quantitation strategy is well-reported in similar subcellular proteomic studies, recent subcellular proteomic studies have demonstrated comparable depth and resolution of proteome data while reducing cost, manual handling, and avoiding the issue of labeling inefficiency and labeling bias (31, 67, 88). This DIA-based approach also reduces effects of missing values and dynamic range limitations comparing to data-dependent acquisition MS (37, 40). Recently, Schessner et al. have applied a DIA-centric approach in subcellular proteomics profiling with demarcated depth, speed, and simplicity (31).

To delineate major cardiac organelle and suborganelle proteome patterns, we included 450 heart-centric protein markers across the majority of organelle and suborganelle niches, including cardiac-specific myofibrils. Our manual curation of cardiac-centric tissue protein markers addressed variations in organellar fractionation profiles observed in different tissues reported (44), and important for constructing highly reliable ML models. Further, incorporating different ML algorithms in our workflow was performed to overcome potential inconsistent predictions of relying on a single analysis algorithm, due to the differences in mathematical principles across algorithms and ML pipelines (94). We have used multiple supervised ML algorithms, including hyperplane-based SVM and decision tree–based RF and XGB, and selected both top performing algorithms for subcellular proteome mapping. It is worth noting that we observed a better performance of SVM- and RF-based subcellular protein classification in comparison to XGB. It is because the current pRolocExtra package only supports the tuning of two parameters (gamma, max depth) of XGB by default. More comprehensive tuning on the different parameters of XGB may be required to increase its performance. Another limitation with our ML approach is that we are only able to identify the predominant subcellular location of a protein but limited in predicting proteins with multisubcellular localizations (95). With more than 50% of the human proteome constituted with multilocalizing proteins (95), this remains a significant informatic and biological challenge to deconvolute different proteins and their subcellular location(s) (58, 64, 96). Incorporation of multilocalizing protein features in data analysis using strategies such as applying multilabel algorithms in imaging-based subcellular protein annotation (97) or using Bayesian-based framework can be applied to account for the uncertainties in multilocalizing proteins (58).

Here, the application of systemic analysis of subcellular proteome from heart provides key understanding of the molecular composition of different subcellular niches in parallel. This molecular systems approach has clear advantage regarding analytical throughput in studying multiple organelles, as well as preserving the spatial information of proteins in their environment. Further, the application of this workflow is particularly promising in deciphering moonlighting proteins, protein sorting and trafficking events, and altered protein subcellular distribution under physiological (age, adaptive cardiac remodeling) or pathophysiological changes of a biological system (98). Primarily, findings from this study impact the subcellular resolution to enable new insights into intracellular signaling events, including at specific time points and the ability to analyze global subcellular proteome dynamics (44). In addition, our pipeline could be combined with affinity-based visual proteomic tools to assess labeled protein changes in subcellular localization and abundance upon perturbation, including failing heart (78, 99). While our pipeline has demonstrated successful separation of different subcellular niches based on the protein intensity profiles of 11 subcellular fractions, we could not provide subcellular proteome map of different cardiac anatomical regions (i.e. atria versus ventricle) or different cell types (i.e. cardiomyocyte versus cardiac fibroblast) due to the low input material from mouse heart. Reducing sample input or reducing fractionation resolution would hinder the proteome depth and reproducibility, feature/niche assignment, and accuracy of derived subcellular proteome data. In addition, although approaches like fluorescence-activated cell sorting could provide cell type–specific insights in subcellular niches, these methods alter the native cardiac environment leading to less representative subcellular proteome landscape such as cell surface epitopes caused by tissue dissociation (100, 101, 102). Future directions will focus on deconvoluting region- or cell type–specific subcellular proteome map using different model organisms, parallel sequential fractionation strategies, multiplex labeling, and higher sensitivity MS technology.

Our study has further implications in quantifying the protein composition of multiple organelles in the heart, including underrepresented organelles such as endosomes and ribosomes. This pipeline can be extended to understand cardiac remodeling relating to altered protein subcellular distribution, such as in response to metabolic or cardiac activity/stress (83, 84, 103). Further, nucleocytoplasmic shuttling of various transcriptional factors occurs to impact cardiac functions. Therefore, our workflow has further implications as a platform to further understand organelle composition and interaction networks in a quantitative and holistic manner. Collectively, our work expands upon previous findings by providing an unprecedented proteomic resource of the subcellular niches of mouse heart. Although studies have employed bioinformatics approaches to annotate the subcellular location of proteins in heart (48, 104), this study integrates *in situ* and *in silico* protein subcellular annotation. We provide a significant update in the depth, coverage, and assignment of cardiac-specific features from mouse heart (27). This high-resolution workflow serves as a draft map of subcellular proteome of mouse heart. This comprehensive framework, which combined multimodal data and integrated knowledge-based and unsupervised annotations, has the power to drive cardiac niche discovery and can be applied to other tissues in health and disease.

## Data Availability

The mass spectrometry proteomics data is deposited to the ProteomeXchange Consortium via the MassIVE partner repository and available via MassIVE (MSV000096615).

## Supplemental data

This article contains supplemental data.

## Conflict of interest

D. W. G. is senior editor for Proteomics (Systems Biology) and Journal of Extracellular Vesicles. His association with these journals did not impact the editorial review or the decision to publish this article. All other authors declare that they have no conflicts of interest with the contents of this article.

## Figures and Tables

**1:**
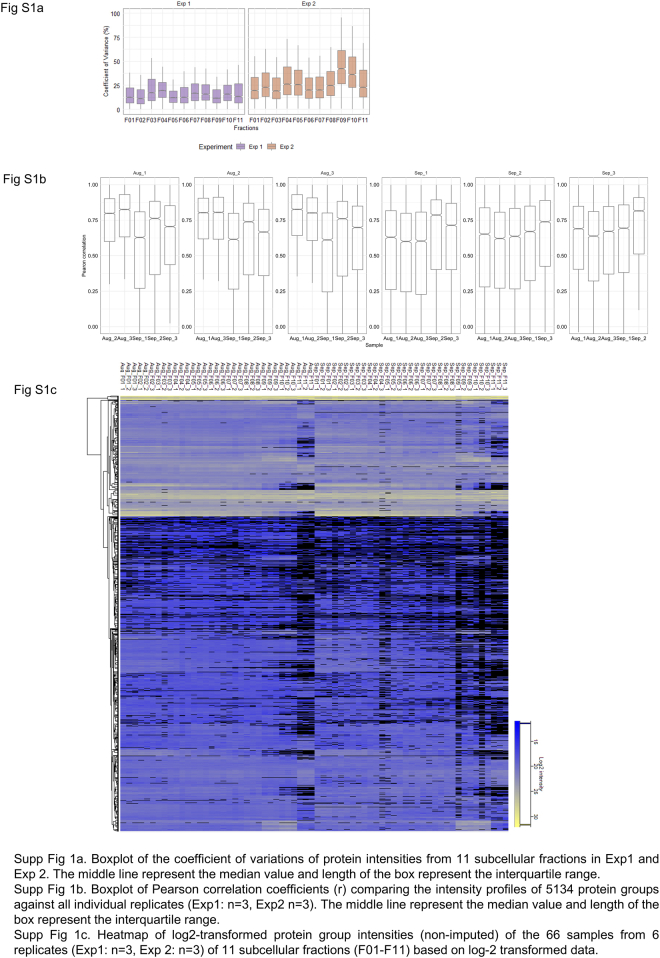
.TIF

**2:**
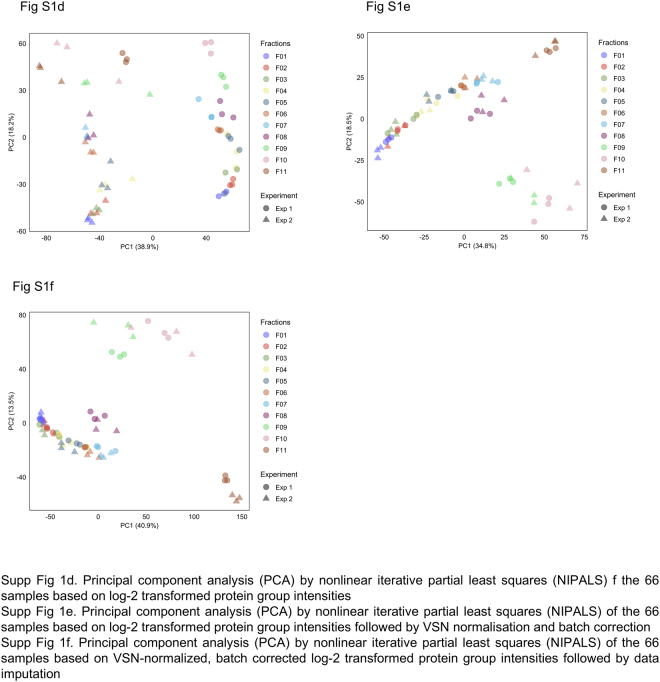
.TIF

**3:**
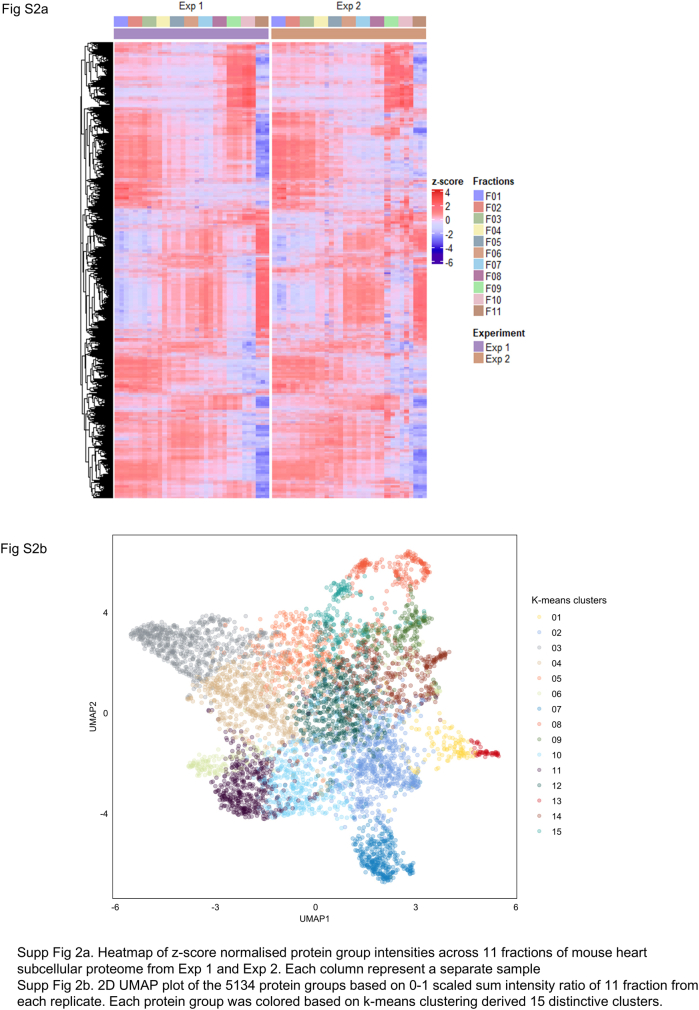
.TIF

**4:**
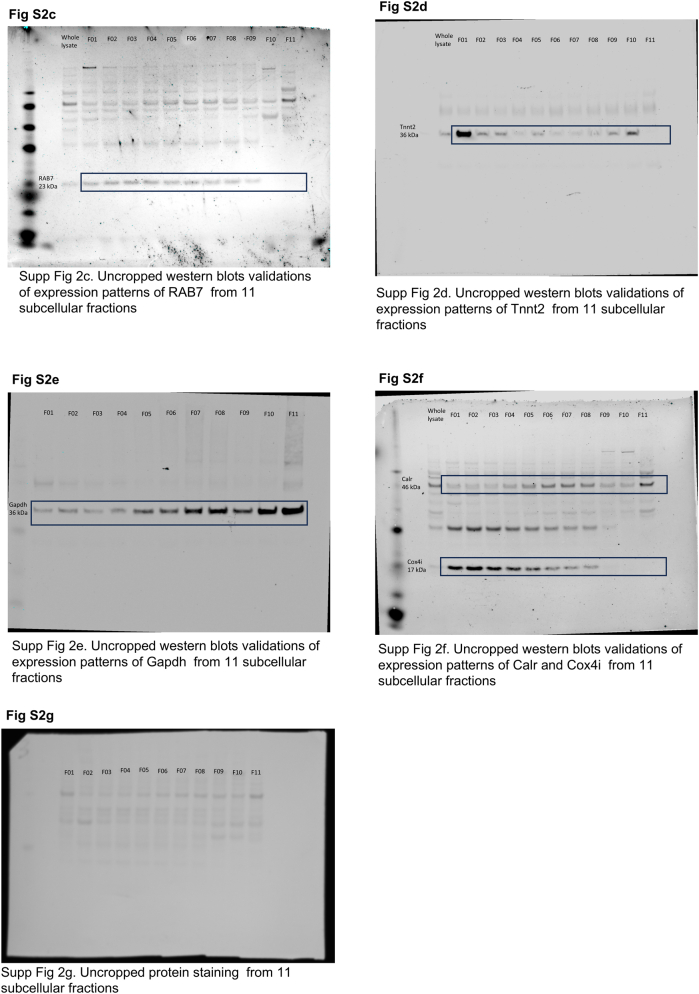
A.TIF

**5:**
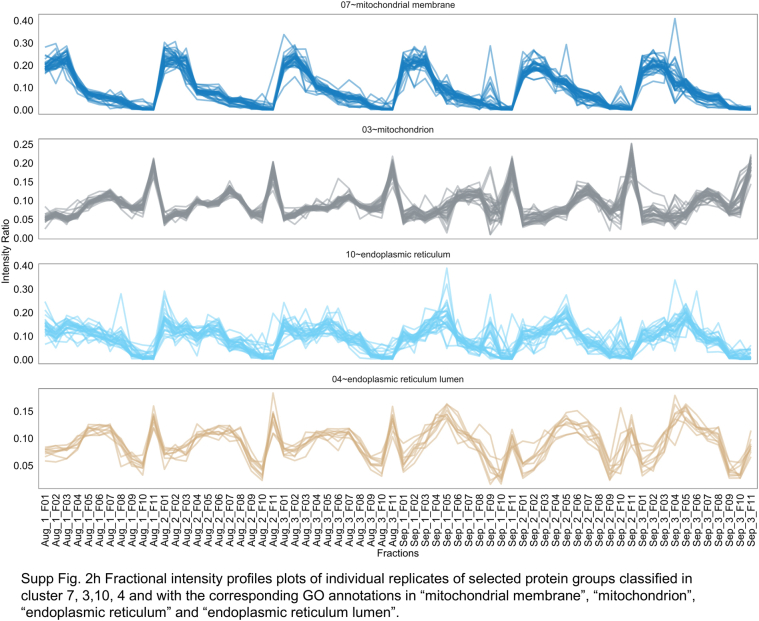
A.TIF

**6:**
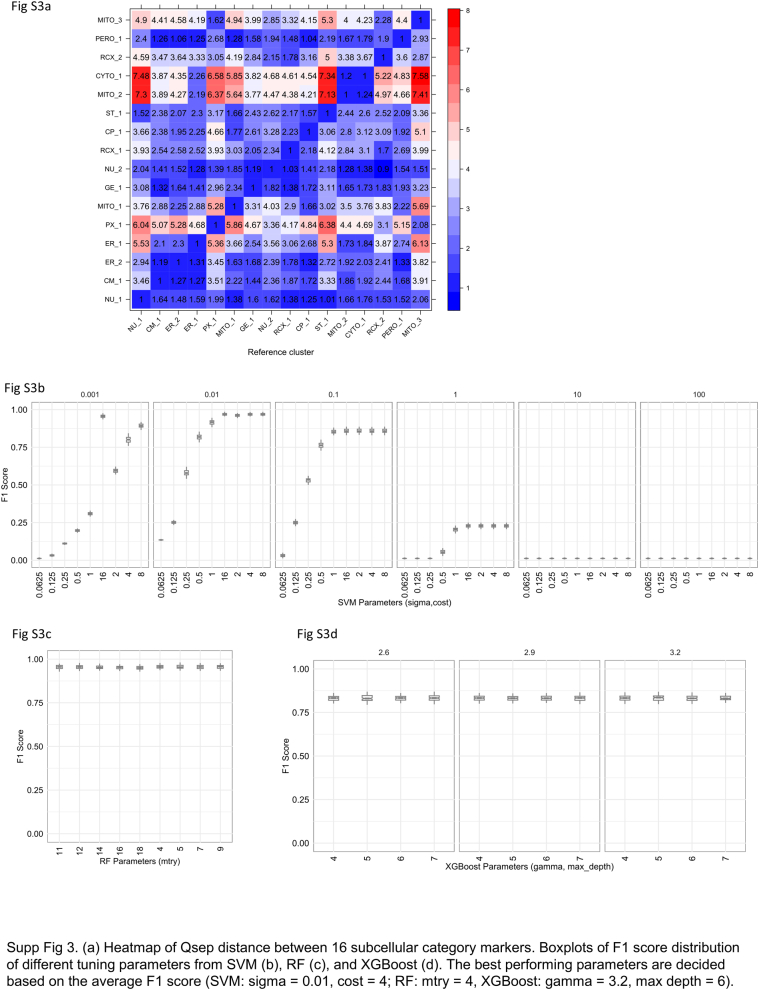
.TIF

**7:**
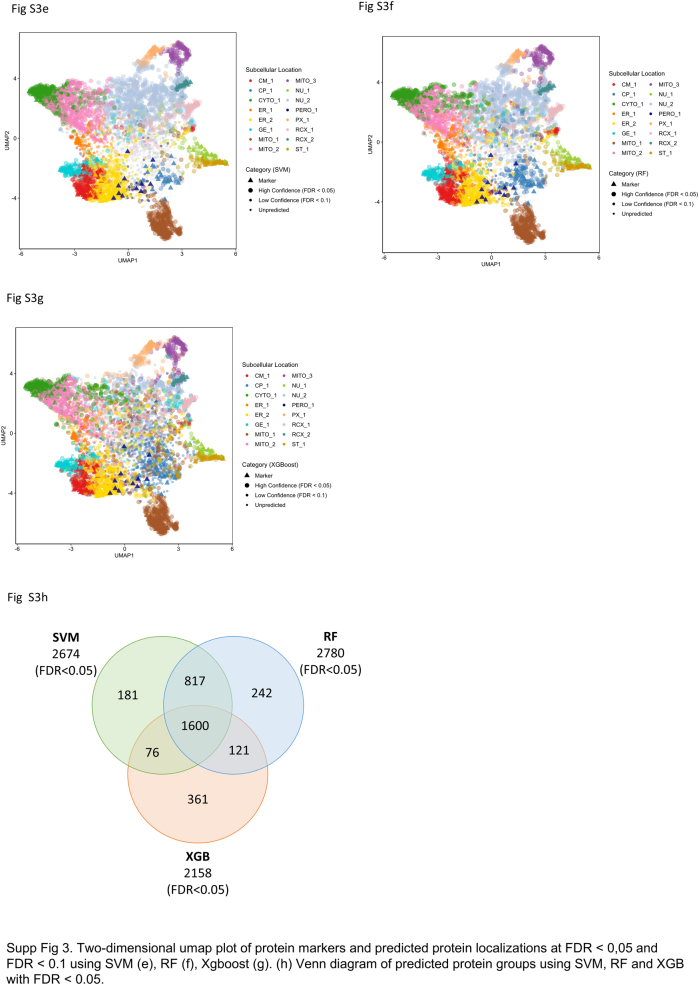
.TIF

**Slide 8:**
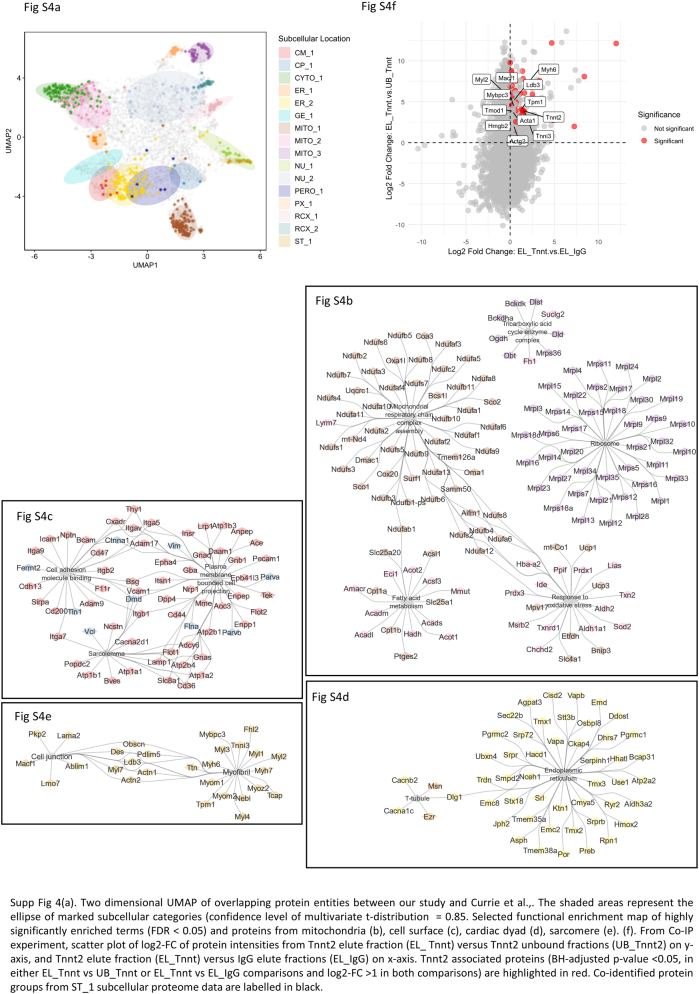


**Supp 4g-k:**
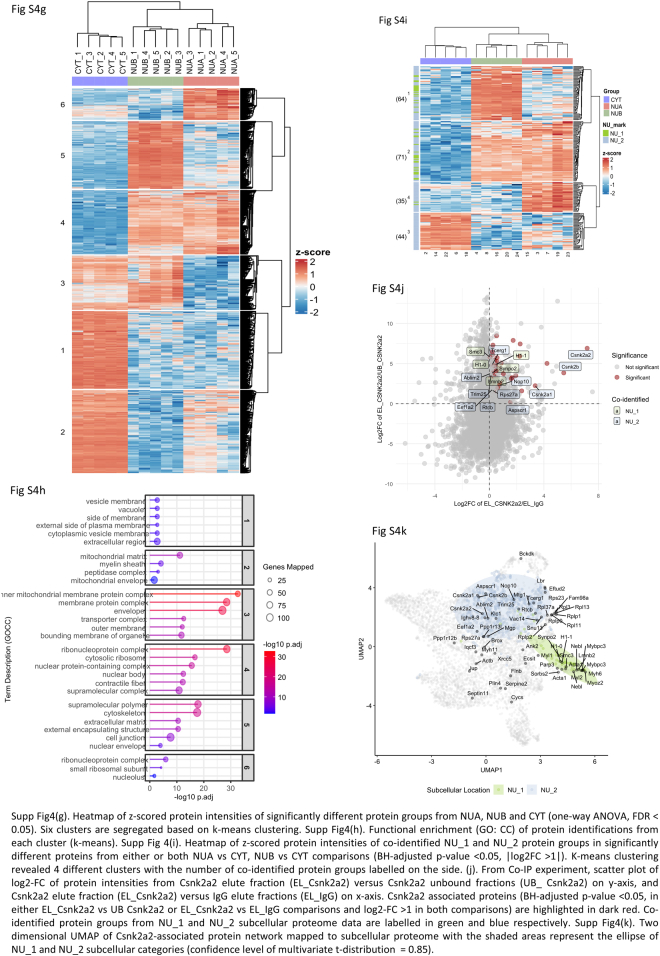


## Supplementary Data

Supp Tables_ALL_07022025_done

## References

[1] Kornienko J., Rodríguez-Martínez M., Fenzl K., Hinze F., Schraivogel D., Grosch M. (2023). Mislocalization of pathogenic RBM20 variants in dilated cardiomyopathy is caused by loss-of-interaction with Transportin-3. Nat. Commun..

[2] Leone M., Musa G., Engel F.B. (2018). Cardiomyocyte binucleation is associated with aberrant mitotic microtubule distribution, mislocalization of RhoA and IQGAP3, as well as defective actomyosin ring anchorage and cleavage furrow ingression. Cardiovasc. Res..

[3] Helmstadter K.G., Ljubojevic-Holzer S., Wood B.M., Taheri K.D., Sedej S., Erickson J.R. (2021). CaMKII and PKA-dependent phosphorylation co-regulate nuclear localization of HDAC4 in adult cardiomyocytes. Basic Res. Cardiol..

[4] Lopez-Crisosto C., Pennanen C., Vasquez-Trincado C., Morales P.E., Bravo-Sagua R., Quest A.F.G. (2017). Sarcoplasmic reticulum–mitochondria communication in cardiovascular pathophysiology. Nat. Rev. Cardiol..

[5] Chen Q., Samidurai A., Thompson J., Hu Y., Das A., Willard B. (2020). Endoplasmic reticulum stress-mediated mitochondrial dysfunction in aged hearts. Biochim. Biophys. Acta (BBA) - Mol. Basis Dis..

[6] Zhao M., Lian A., Zhong L., Guo R. (2022). The regulatory mechanism between lysosomes and mitochondria in the aetiology of cardiovascular diseases. Acta Physiol..

[7] Zhao G., Qiu Y., Zhang H.M., Yang D. (2019). Intercalated discs: cellular adhesion and signaling in heart health and diseases. Heart Fail Rev..

[8] Dhalla N.S., Saini-Chohan H.K., Rodriguez-Leyva D., Elimban V., Dent M.R., Tappia P.S. (2009). Subcellular remodelling may induce cardiac dysfunction in congestive heart failure. Cardiovasc. Res..

[9] Tappia P.S., Shah A.K., Ramjiawan B., Dhalla N.S. (2022). Modification of ischemia/reperfusion-induced alterations in subcellular organelles by ischemic preconditioning. Int. J. Mol. Sci..

[10] Dhalla N.S., Takeda N., Rodriguez-Leyva D., Elimban V. (2014). Mechanisms of subcellular remodeling in heart failure due to diabetes. Heart Fail Rev..

[11] Bhullar S.K., Shah A.K., Dhalla N.S. (2021). Role of angiotensin II in the development of subcellular remodeling in heart failure. Open Exploration.

[12] Gabr R.E., El-Sharkawy A.M.M., Schär M., Panjrath G.S., Gerstenblith G., Weiss R.G. (2018). Cardiac work is related to creatine kinase energy supply in human heart failure: a cardiovascular magnetic resonance spectroscopy study. J. Cardiovasc. Magn. Reson..

[13] Neubauer S. (2007). The failing heart — an engine out of fuel. N. Engl. J. Med..

[14] Ramalingam A., Budin S.B., Mohd Fauzi N., Ritchie R.H., Zainalabidin S. (2021). Targeting mitochondrial reactive oxygen species-mediated oxidative stress attenuates nicotine-induced cardiac remodeling and dysfunction. Sci. Rep..

[15] Paradies G., Petrosillo G., Pistolese M., Di Venosa N., Federici A., Ruggiero F.M. (2004). Decrease in mitochondrial complex I activity in ischemic/reperfused rat heart: involvement of reactive oxygen species and cardiolipin. Circ. Res..

[16] Tajes M., Díez-López C., Enjuanes C., Moliner P., Ferreiro J.L., Garay A. (2021). Neurohormonal activation induces intracellular iron deficiency and mitochondrial dysfunction in cardiac cells. Cell Biosci..

[17] Li L., Thompson J., Hu Y., Lesnefsky E.J., Willard B., Chen Q. (2022). Calpain-mediated protein targets in cardiac mitochondria following ischemia–reperfusion. Sci. Rep..

[18] Yang Y., Li J., Han T.L., Zhou X., Qi H., Baker P.N. (2020). Endoplasmic reticulum stress may activate NLRP3 inflammasomes via TXNIP in preeclampsia. Cell Tissue Res..

[19] Jiang L., Qiao Y., Wang Z., Ma X., Wang H., Li J. (2020). Inhibition of microRNA-103 attenuates inflammation and endoplasmic reticulum stress in atherosclerosis through disrupting the PTEN-mediated MAPK signaling. J. Cell Physiol..

[20] Liu Z., Zhao N., Zhu H., Zhu S., Pan S., Xu J. (2015). Circulating interleukin-1β promotes endoplasmic reticulum stress-induced myocytes apoptosis in diabetic cardiomyopathy via interleukin-1 receptor-associated kinase-2. Cardiovasc. Diabetol..

[21] Tam A.B., Roberts L.S., Chandra V., Rivera I.G., Nomura D.K., Forbes D.J. (2018). The UPR activator ATF6 responds to proteotoxic and lipotoxic stress by distinct mechanisms. Dev. Cell.

[22] Shao X., Meng C., Song W., Zhang T., Chen Q. (2023). Subcellular visualization: organelle-specific targeted drug delivery and discovery. Adv. Drug Deliv. Rev..

[23] Ayagama T., Bose S.J., Capel R.A., Priestman D.A., Berridge G., Fischer R. (2021). A modified density gradient proteomic-based method to analyze endolysosomal proteins in cardiac tissue. iScience.

[24] McLaughlin K.L., Hagen J.T., Coalson H.S., Nelson M.A.M., Kew K.A., Wooten A.R. (2020). Novel approach to quantify mitochondrial content and intrinsic bioenergetic efficiency across organs. Sci. Rep..

[25] Lu F., Ma Q., Xie W., Liou C.L., Zhang D., Sweat M.E. (2022). CMYA5 establishes cardiac dyad architecture and positioning. Nat. Commun..

[26] Rudolph F., Fink C., Hüttemeister J., Kirchner M., Radke M.H., Lopez Carballo J. (2020). Deconstructing sarcomeric structure–function relations in titin-BioID knock-in mice. Nat. Commun..

[27] Kislinger T., Cox B., Kannan A., Chung C., Hu P., Ignatchenko A. (2006). Global survey of organ and organelle protein expression in mouse: combined proteomic and transcriptomic profiling. Cell.

[28] Foster L.J., de Hoog C.L., Zhang Y., Zhang Y., Xie X., Mootha V.K. (2006). A mammalian organelle map by protein correlation profiling. Cell.

[29] Geladaki A., Kočevar Britovšek N., Breckels L.M., Smith T.S., Vennard O.L., Mulvey C.M. (2019). Combining LOPIT with differential ultracentrifugation for high-resolution spatial proteomics. Nat. Commun..

[30] Mulvey C.M., Breckels L.M., Geladaki A., Britovšek N.K., Nightingale D.J.H., Christoforou A. (2017). Using hyperLOPIT to perform high-resolution mapping of the spatial proteome. Nat. Protoc..

[31] Schessner J.P., Albrecht V., Davies A.K., Sinitcyn P., Borner G.H.H. (2023). Deep and fast label-free dynamic organellar mapping. Nat. Commun..

[32] Itzhak D.N., Tyanova S., Cox J., Borner G.H.H. (2016). Global, quantitative and dynamic mapping of protein subcellular localization. Elife.

[33] Itzhak D.N., Davies C., Tyanova S., Mishra A., Williamson J., Antrobus R. (2017). A mass spectrometry-based approach for mapping protein subcellular localization reveals the spatial proteome of mouse primary neurons. Cell Rep..

[34] Arslan T., Pan Y., Mermelekas G., Vesterlund M., Orre L.M., Lehtiö J. (2022). SubCellBarCode: integrated workflow for robust spatial proteomics by mass spectrometry. Nat. Protoc..

[35] Claridge B., Rai A., Lees J.G., Fang H., Lim S.Y., Greening D.W. (2023). Cardiomyocyte intercellular signalling increases oxidative stress and reprograms the global- and phospho-proteome of cardiac fibroblasts. J. Extracellular Biol..

[36] Lagundžin D., Krieger K.L., Law H.C.H., Woods N.T. (2022). An optimized co-immunoprecipitation protocol for the analysis of endogenous protein-protein interactions in cell lines using mass spectrometry. STAR Protoc..

[37] Cross J., Rai A., Fang H., Claridge B., Greening D.W. (2023). Rapid and in-depth proteomic profiling of small extracellular vesicles for ultralow samples. Proteomics.

[38] Hughes C.S., Moggridge S., Müller T., Sorensen P.H., Morin G.B., Krijgsveld J. (2019). Single-pot, solid-phase-enhanced sample preparation for proteomics experiments. Nat. Protoc..

[39] Demichev V., Messner C.B., Vernardis S.I., Lilley K.S., Ralser M. (2019). DIA-NN: neural networks and interference correction enable deep proteome coverage in high throughput. Nat. Methods.

[40] Fang H., Greening D.W. (2023). An optimized data-independent acquisition strategy for comprehensive analysis of human plasma proteome. Methods Mol. Biol..

[41] Bateman A., UniProt Consortium (2021). UniProt: the universal protein knowledgebase in 2021. Nucleic Acids Res..

[42] Čuklina J., Lee C.H., Williams E.G., Sajic T., Collins B.C., Rodríguez Martínez M. (2021). Diagnostics and correction of batch effects in large-scale proteomic studies: a tutorial. Mol. Syst. Biol..

[43] Ritchie M.E., Phipson B., Wu D., Hu Y., Law C.W., Shi W. (2015). Limma powers differential expression analyses for RNA-sequencing and microarray studies. Nucleic Acids Res..

[44] Martinez-Val A., Bekker-Jensen D.B., Steigerwald S., Koenig C., Østergaard O., Mehta A. (2021). Spatial-proteomics reveals phospho-signaling dynamics at subcellular resolution. Nat. Commun..

[45] Gatto L., Breckels L.M., Mulvey C.M., Lilley K.S. (2018). A Bioconductor workflow for processing and analysing spatial proteomics data. F1000Research.

[46] GERAULT M.-A. (2023).

[47] Go C.D., Knight J.D.R., Rajasekharan A., Rathod B., Hesketh G.G., Abe K.T. (2021). A proximity-dependent biotinylation map of a human cell. Nat..

[48] Lee S.H., Hadipour-Lakmehsari S., Kim D.H., Di Paola M., Kuzmanov U., Shah S. (2020). Bioinformatic analysis of membrane and associated proteins in murine cardiomyocytes and human myocardium. Sci. Data.

[49] Thul P.J., Lindskog C. (2018). The human protein atlas: a spatial map of the human proteome. Protein Sci..

[50] Bateman A., UniProt Consortium (2023). UniProt: the universal protein knowledgebase in 2023. Nucleic Acids Res..

[51] Baldarelli R.M., Smith C.L., Ringwald M., Richardson J.E., Bult C.J., Mouse Genome Informatics Group (2024). Mouse Genome Informatics: an integrated knowledgebase system for the laboratory mouse. Genetics.

[52] Carbon S., The Gene Ontology Consortium (2019). The gene ontology resource: 20 years and still GOing strong. Nucleic Acids Res..

[53] Watson J., Smith M., Francavilla C., Schwartz J.M. (2022). SubcellulaRVis: a web-based tool to simplify and visualise subcellular compartment enrichment. Nucleic Acids Res..

[54] Szklarczyk D., Kirsch R., Koutrouli M., Nastou K., Mehryary F., Hachilif R. (2023). The STRING database in 2023: protein–protein association networks and functional enrichment analyses for any sequenced genome of interest. Nucleic Acids Res..

[55] Gu Z., Eils R., Schlesner M. (2016). Complex heatmaps reveal patterns and correlations in multidimensional genomic data. Bioinformatics.

[56] Wickham H. (2016).

[57] Wang B., Zhang X., Xu C., Han X., Wang Y., Situ C. (2023). DeepSP: a deep learning framework for spatial proteomics. J. Proteome Res..

[58] Crook O.M., Mulvey C.M., Kirk P.D.W., Lilley K.S., Gatto L. (2018). A Bayesian mixture modelling approach for spatial proteomics. PLoS Comput. Biol..

[59] Yu G., Wang L.-G., Han Y., He Q.-Y. (2012). clusterProfiler: an R package for comparing biological themes among gene clusters. OMICS.

[60] Doncheva N.T., Morris J.H., Holze H., Kirsch R., Nastou K.C., Cuesta-Astroz Y. (2023). Cytoscape stringApp 2.0: analysis and visualization of heterogeneous biological networks. J. Proteome Res..

[61] Crook O.M., Breckels L.M., Lilley K.S., Kirk P.D.W., Gatto L. (2019). A Bioconductor workflow for the Bayesian analysis of spatial proteomics. F1000Research.

[62] Giansanti P., Samaras P., Bian Y., Meng C., Coluccio A., Frejno M. (2022). Mass spectrometry-based draft of the mouse proteome. Nat. Methods.

[63] Lu T., Qian L., Xie Y., Zhang Q., Liu W., Ge W. (2022). Tissue-characteristic expression of mouse proteome. Mol. Cell Proteomics.

[64] Currie J., Manda V., Robinson S.K., Lai C., Agnihotri V., Hidalgo V. (2024). Simultaneous proteome localization and turnover analysis reveals spatiotemporal features of protein homeostasis disruptions. Nat. Commun..

[65] Miller M.R., Sarah S., Foster B. (2016). Manual of Cardiovascular Proteomics.

[66] Lee S.H., Park D.J., Yun W.S., Park J.E., Choi J.S., Key J. (2020). Endocytic trafficking of polymeric clustered superparamagnetic iron oxide nanoparticles in mesenchymal stem cells. J. Controlled Release.

[67] Kandigian S.E., Ethier E.C., Kitchen R.R., Lam T.T., Arnold S.E., Carlyle B.C. (2022). Proteomic characterization of post-mortem human brain tissue following ultracentrifugation-based subcellular fractionation. Brain Commun..

[68] Ohta S., Bukowski-Wills J.C., Sanchez-Pulido L., Alves F.d.L., Wood L., Chen Z.A. (2010). The protein composition of mitotic chromosomes determined using multiclassifier combinatorial proteomics. Cell.

[69] Torun F.M., Virreira Winter S., Doll S., Riese F.M., Vorobyev A., Mueller-Reif J.B. (2023). Transparent exploration of machine learning for biomarker discovery from proteomics and omics data. J. Proteome Res..

[70] Unterhuber M., Kresoja K.P., Rommel K.P., Besler C., Baragetti A., Klöting N. (2021). Proteomics-enabled deep learning machine algorithms can enhance prediction of mortality. J. Am. Coll. Cardiol..

[71] Park I., Kim K.e., Kim J., Kim A.K., Bae S., Jung M. (2023). Mitochondrial matrix RTN4IP1/OPA10 is an oxidoreductase for coenzyme Q synthesis. Nat. Chem. Biol..

[72] Busch J.D., Cipullo M., Atanassov I., Bratic A., Silva Ramos E., Schöndorf T. (2019). MitoRibo-tag mice provide a tool for in vivo studies of mitoribosome composition. Cell Rep..

[73] Liu G., Papa A., Katchman A.N., Zakharov S.I., Roybal D., Hennessey J.A. (2020). Mechanism of adrenergic CaV1.2 stimulation revealed by proximity proteomics. Nat..

[74] Quick A.P., Wang Q., Philippen L.E., Barreto-Torres G., Chiang D.Y., Beavers D. (2017). SPEG (striated muscle preferentially expressed protein kinase) is essential for cardiac function by regulating junctional membrane complex activity. Circ. Res..

[75] Ladha F.A., Thakar K., Pettinato A.M., Legere N., Ghahremani S., Cohn R. (2021). Actinin BioID reveals sarcomere crosstalk with oxidative metabolism through interactions with IGF2BP2. Cell Rep..

[76] Soni S., Raaijmakers A.J.A., Raaijmakers L.M., Damen J.M.A., van Stuijvenberg L., Vos M.A. (2016). A proteomics approach to identify new putative cardiac intercalated disk proteins. PLoS One.

[77] Li Y., Merkel C.D., Zeng X., Heier J.A., Cantrell P.S., Sun M. (2019). The N-cadherin interactome in primary cardiomyocytes as defined using quantitative proximity proteomics. J. Cell Sci..

[78] Berg Luecke L., Waas M., Littrell J., Wojtkiewicz M., Castro C., Burkovetskaya M. (2023). Surfaceome mapping of primary human heart cells with CellSurfer uncovers cardiomyocyte surface protein LSMEM2 and proteome dynamics in failing hearts. Nat. Cardiovasc. Res..

[79] Sharma P., Abbasi C., Lazic S., Teng A.C.T., Wang D., Dubois N. (2015). Evolutionarily conserved intercalated disc protein Tmem65 regulates cardiac conduction and connexin 43 function. Nat. Commun..

[80] Bertero A., Rosa-Garrido M. (2021). Three-dimensional chromatin organization in cardiac development and disease. J. Mol. Cell Cardiol..

[81] Cheedipudi S.M., Matkovich S.J., Coarfa C., Hu X., Robertson M.J., Sweet M. (2019). Genomic reorganization of lamin-associated domains in cardiac myocytes is associated with differential gene expression and DNA methylation in human dilated cardiomyopathy. Circ. Res..

[82] Silk E., Zhao H., Weng H., Ma D. (2017). The role of extracellular histone in organ injury. Cell Death Dis..

[83] Fiordelisi A., Iaccarino G., Morisco C., Coscioni E., Sorriento D. (2019). NFkappaB is a key player in the crosstalk between inflammation and cardiovascular diseases. Int. J. Mol. Sci..

[84] Vashi R., Patel B.M. (2020). NRF2 in cardiovascular diseases: a ray of hope. J. Cardiovasc. Translational Res..

[85] Bekeredjian R., Walton C.B., MacCannell K.A., Ecker J., Kruse F., Outten J.T. (2010). Conditional HIF-1α expression produces a reversible cardiomyopathy. PLoS One.

[86] Safabakhsh S., Sar F., Martelotto L., Haegert A., Singhera G., Hanson P. (2022). Isolating nuclei from frozen human heart tissue for single-nucleus RNA sequencing. Curr. Protoc..

[87] Christoforou A., Mulvey C.M., Breckels L.M., Geladaki A., Hurrell T., Hayward P.C. (2016). A draft map of the mouse pluripotent stem cell spatial proteome. Nat. Commun..

[88] Krahmer N., Najafi B., Schueder F., Quagliarini F., Steger M., Seitz S. (2018). Organellar proteomics and phospho-proteomics reveal subcellular reorganization in diet-induced hepatic steatosis. Dev. Cell.

[89] Barylyuk K., Koreny L., Ke H., Butterworth S., Crook O.M., Lassadi I. (2020). A comprehensive subcellular atlas of the toxoplasma proteome via hyperLOPIT provides spatial context for protein functions. Cell Host Microbe..

[90] Christopher J.A., Breckels L.M., Crook O.M., Vazquez-Chantada M., Barratt D., Lilley K.S. (2024). Global proteomics indicates subcellular-specific anti-ferroptotic responses to ionizing radiation. Mol. Cell Proteomics.

[91] Zeng X., Wang H., Xing X., Wang Q., Li W. (2016). Dexmedetomidine protects against transient global cerebral ischemia/reperfusion induced oxidative stress and inflammation in diabetic rats. PLoS One.

[92] Jin J.K., Whittaker R., Glassy M.S., Barlow S.B., Gottlieb R.A., Glembotski C.C. (2008). Localization of phosphorylated αB-crystallin to heart mitochondria during ischemia-reperfusion. Am. J. Physiol. Heart Circ. Physiol..

[93] Lau E., Cao Q., Ng D.C.M., Bleakley B.J., Dincer T.U., Bot B.M. (2016). A large dataset of protein dynamics in the mammalian heart proteome. Sci. Data.

[94] Mou M., Pan Z., Lu M., Sun H., Wang Y., Luo Y. (2022). Application of machine learning in spatial proteomics. J. Chem. Inf. Model.

[95] Thul P.J., Åkesson L., Wiking M., Mahdessian D., Geladaki A., Ait Blal H. (2017). A subcellular map of the human proteome. Science.

[96] Crook O.M., Davies C.T.R., Breckels L.M., Christopher J.A., Gatto L., Kirk P.D.W. (2022). Inferring differential subcellular localisation in comparative spatial proteomics using BANDLE. Nat. Commun..

[97] Wang F., Wei L. (2022). Multi-scale deep learning for the imbalanced multi-label protein subcellular localization prediction based on immunohistochemistry images. Bioinformatics.

[98] Christopher J.A., Stadler C., Martin C.E., Morgenstern M., Pan Y., Betsinger C.N. (2021). Subcellular proteomics. Nat. Rev. Methods Primers.

[99] Reicher A., Reiniš J., Ciobanu M., Růžička P., Malik M., Siklos M. (2024). Pooled multicolour tagging for visualizing subcellular protein dynamics. Nat. Cell Biol..

[100] Mattei D., Ivanov A., van Oostrum M., Pantelyushin S., Richetto J., Mueller F. (2020). Enzymatic dissociation induces transcriptional and proteotype bias in brain cell populations. Int. J. Mol. Sci..

[101] Abuzakouk M., Feighery C., O’Farrelly C. (1996). Collagenase and Dispase enzymes disrupt lymphocyte surface molecules. J. Immunol. Methods.

[102] Hosseini V., Kalantary-Charvadeh A., Hasegawa K., Nazari Soltan Ahmad S., Rahbarghazi R., Mahdizadeh A. (2020). A mechanical non-enzymatic method for isolation of mouse embryonic fibroblasts. Mol. Biol. Rep..

[103] Cortés R., Roselló-Lletí E., Rivera M., Martínez-Dolz L., Salvador A., Azorín I. (2010). Influence of heart failure on nucleocytoplasmic transport in human cardiomyocytes. Cardiovasc. Res..

[104] Doll S., Dreßen M., Geyer P.E., Itzhak D.N., Braun C., Doppler S.A. (2017). Region and cell-type resolved quantitative proteomic map of the human heart. Nat. Commun..

